# The ABCs of hearing and vision care in long-term care communities: a systematic review and behavioral systems map of Actors, Behaviors, and COM-B factors

**DOI:** 10.1093/geront/gnag020

**Published:** 2026-03-14

**Authors:** Divya Anantharaman, Carly Meyer, Mehwish Nisar, Sheela Kumaran, Lisa Keay, Sue McAvoy, Piers Dawes

**Affiliations:** University of Queensland Centre for Hearing Research (CHEAR), School of Health and Rehabilitation Sciences, The University of Queensland, Brisbane, Queensland, Australia; University of Queensland Centre for Hearing Research (CHEAR), School of Health and Rehabilitation Sciences, The University of Queensland, Brisbane, Queensland, Australia; Bolton Clarke Research Institute, Brisbane, Queensland, Australia; University of Queensland Centre for Hearing Research (CHEAR), School of Health and Rehabilitation Sciences, The University of Queensland, Brisbane, Queensland, Australia; School of Optometry and Vision Science, Faculty of Medicine and Health, University of New South Wales, Sydney, New South Wales, Australia; School of Optometry and Vision Science, Faculty of Medicine and Health, University of New South Wales, Sydney, New South Wales, Australia; The George Institute for Global Health, University of New South Wales, Sydney, New South Wales, Australia; The University of Queensland Centre for the Business and Economics of Health, The University of Queensland, Brisbane, Queensland, Australia; University of Queensland Centre for Hearing Research (CHEAR), School of Health and Rehabilitation Sciences, The University of Queensland, Brisbane, Queensland, Australia

**Keywords:** Audiology, Eye, Integrated care, Aged care, Residents

## Abstract

**Background and Objectives:**

Despite the high prevalence of hearing and vision impairment among people living in long-term care (LTC) and their impact on quality of life, these sensory challenges are often disregarded. Hence, this systematic review aims to identify the actors and factors influencing hearing and vision care behavior in LTC and construct a literature informed behavioral systems map to demonstrate this complex system.

**Research Design and Methods:**

A systematic review across five databases yielded 23 articles from 3,644 screened (January 2013–September 2024). Data on sensory care behaviors in LTC (screening, referring, receiving care, device use, and communication adaptation) were extracted, coded, and mapped to the COM-B (Capability, Opportunity, and Motivation—Behavior) framework with associated actors. A behavioral systems mapping prototype was developed with the synthesized data.

**Results:**

Actors included residents, family members, care staff, LTC management, and hearing and vision professionals. Analysis revealed 31 factors across the COM-B framework related to the five sensory care behaviors. Eighteen factors affected multiple behaviors. Most salient among the interconnecting factors were collaborative care, family engagement, infrastructure, and perception of value. The synthesized behavioral systems map revealed ten feedback loops driving sensory care behaviors.

**Discussion and Implications:**

The behavioral systems map provides crucial groundwork for developing comprehensive solutions to enhance hearing and vision care across LTC settings globally. It reveals that hearing and vision care involves multiple stakeholder groups and interconnected COM-B factors, whose components of capability, opportunity and motivation collectively influence care through feedback dynamics, creating emergent, self-organizing behaviors requiring multifaceted approaches.

## Background

Globally, more than 1 in 5 people are aged over 60 years, and around 14% of these people experience functional limitations that limit their capacity to live independently (World Health Organization, 2020, 2022). Long-term care (LTC) has therefore become essential in providing support for older adults experiencing, or at risk of, decline in physical and mental capacity (World Health Organization, 2015, 2022). According to the World Health Organization (WHO), LTC helps older adults maintain a level of functional ability that aligns with their basic rights, fundamental freedoms, and human dignity (World Health Organization, 2015). A crucial aspect of maintaining this functional ability is a person’s hearing and vision (Heine et al., 2019; Tseng et al., 2018). Hearing and vision functioning relates to several aspects of healthy living, including communication and social engagement, mental well-being, mobility, and the ability to perform basic and instrumental activities of daily living (Gopinath et al., 2021; Guthrie et al., 2018; Heine et al., 2019). A longitudinal study reported that older adults (>60 years) with visual or bilateral hearing impairment had a 37% reduction in the likelihood of successful aging (defined as absence of disease, physical and mental disability) over five years (Gopinath et al., 2021). Despite this, hearing and vision care is frequently disregarded in LTCs. Several studies explored staff-related barriers including time constraints, lack of knowledge about hearing or vision care, insufficient collaborative support from colleagues and managers, low prioritization of hearing or vision care, and beliefs such as eye conditions cannot be improved or hearing aids do not benefit people living with dementia (Bott et al., 2022; Cross et al., 2023a; Dawes et al., 2021; Kergoat et al., 2014). In addition to staff, residents, family members, and hearing and vision professionals contribute to the gaps in the provision of hearing and vision support (Anantharaman et al., 2025; Bowen et al., 2016).

Previous studies typically investigated the provision of hearing and vision support in LTC at the level of individual stakeholders (Cross et al., 2023a, 2025; Kergoat et al., 2014; Marmamula et al., 2023b). As LTC is a complex system, a focus on individual stakeholders’ behaviors may not be sufficient. Improving hearing and vision care behavior requires multifaceted approaches that address both the barriers and enablers faced by multiple stakeholders across the wider social infrastructure of LTC. However, to our knowledge, no studies have modelled barriers and facilitators to carrying out hearing and vision care behaviors at a system level. Behavioral systems mapping (BSM) is a recent approach in behavioral science that helps understand and change human behavior in complex systems. It works by identifying actors, behaviors, and influences on behavior, as well as mapping the relationships between them. BSM creates visual representations that show what causes current behaviors, providing insights into what needs changing to improve the system. These maps can be further enhanced by connecting identified influences on established behavioral theories. The capability, opportunity, and motivation model of behavior (COM-B) (Michie et al., 2011) is a widely used behavioral science framework that integrates well with systems mapping because it conceptualizes these elements within the interactive system (Allison et al., 2024; Davan Wetton et al., 2025; Hale et al., 2022; West, 2020). It has frequently been applied in healthcare settings, including LTC, where it has been used to improve staff practices and patient care (McCarthy et al., 2022; Michie et al., 2014). Applying this framework ensures a structured, evidence-based approach to mapping behavioral influences in hearing and vision care. Systems thinking approaches are used and recommended by the WHO and the United Kingdom’s Government Office for Science to strengthen the health and social care systems (Allison et al., 2024; De Savigny & Adam, 2009; Hale et al., 2022; The Government Office for Science, 2022; West, 2020). Hence, we aimed to i) systematically review the literature to identify the actors involved, and synthesize the factors influencing, the behaviors related to hearing and vision care in LTC; and ii) to develop a literature informed behavioral systems map for understanding the interactions and interdependencies of actors and factors influencing hearing and vision care behaviors in LTC.

## Methods

We used systematic evidence synthesis to derive a behavioral systems map for vision and hearing care in LTC (Littlejohns et al., 2021). Our methodology integrated systematic review techniques with BSM informed by the COM-B framework (Michie et al., 2011). We first conducted a systematic review to synthesize existing knowledge. We identified key actors and factors influencing hearing and vision care behaviors, which were then mapped to the COM-B framework. Finally, we developed a BSM to illustrate the interconnected relationships between these elements.

The systematic review protocol was registered in PROSPERO (CRD42023473031), the International Prospective Register of Systematic Reviews, and followed the 2020 PRISMA (Preferred Reporting Items for Systematic Reviews and Meta-Analyses) guidelines. The **Population/Problem (P)** included either i) people living in LTC with hearing or vision loss or both, or ii) people engaged in hearing and vision-related support for residents in LTC (informal caregivers or family members of residents, aged care staff, and hearing and vision professionals). The **Phenomenon of Interest (I)** was any actor (referring to individual, group or community) and factors (referring to any variable capable of exerting positive or negative influence) influencing behaviors (referring to actions, activities or reactions/responses) related to hearing and vision care including (i) screening for hearing and vision problems among residents (ii) referring residents to hearing and vision professionals including audiologists, otorhinolaryngologists, Ear, Nose, and Throat specialists, optometrists, and ophthalmologists (iii) residents receiving hearing and vision care from health care professionals, including attending appointments, assessment, and diagnosis, (iv) residents’ use of hearing or vision devices (i.e., hearing aids, cochlear implants, spectacles, magnifying glasses, low vision aids, or assistive software), (v) communicating with residents with hearing and/or vision loss. The **Context (Co)** was LTC communities. These were identified as communities that provide room and board, manage chronic health conditions, and offer varying degrees of 24-hour functional support and assistance with activities of daily living (ADL) for individuals with physical and/or cognitive impairment (Sanford et al., 2015).

### Search strategy

The search was conducted in six electronic databases: Cochrane Database of Systematic Reviews, PubMed, CINAHL/EBSCO, EMBASE, APA PsycINFO, and Web of Science. Search terms were developed with the guidance of an experienced health science librarian at the University. A combination of Medical Subject Heading terms, keywords, and synonyms for the terms, “hearing”, “vision”, and “long-term care” was included (Appendix 1—see online supplementary material). The search was limited to publications between January 2013 and September 2024, and the retrieved articles were uploaded to the Covidence software and duplicates were removed.

### Study selection and quality assessment

Title, abstract and full-text screening was conducted by two independent reviewers (D.A. and M.N.) based on the criteria below. Reference lists of eligible studies and review articles were manually screened to identify if studies met the inclusion criteria. Any conflicts between the reviewers were resolved through discussion or by the involvement of two additional reviewers (C.M. and P.D.).

Inclusion Criteria: (i) Original, peer-reviewed articles that used mixed, qualitative, or quantitative approaches. (ii) Only articles published in English, due to resource availability.

Exclusion Criteria: (a) Conference abstracts, proceedings, books, or letters. (b) Articles reporting only the prevalence or incidence of hearing or vision impairment in LTC. (c) Articles that did not provide separate findings for LTC. (d) Intervention-based articles (since our objective was to capture the typical practices for providing hearing and vision care in LTCs).

Two independent reviewers (D.A. and M.N.) evaluated the quality of each study using two established tools: (i) the Mixed Methods Appraisal Tool (MMAT, version 2018) by Hong et al. (2018) to assess the methodological quality.(Hong et al., 2018) Based on the study type, the reviewers selected the appropriate criteria category (qualitative, quantitative descriptive, or mixed methods) within the MMAT for each study; (ii) Levels of Evidence (March 2009) was used to assess the strength and reliability of research findings. See online supplementary material Appendix 2 (OCEBM Levels of Evidence Working Group, 2011) for details.

### Data extraction and data synthesis

**Figure 1:**
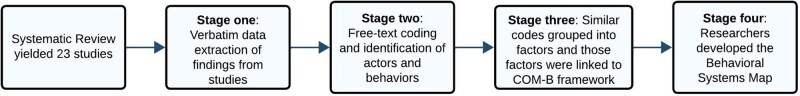
The four-stage process of data extraction, data coding and synthesis, data grouping, and the behavioral systems map development.

We adapted the methods proposed for the thematic synthesis of qualitative research in systematic reviews (Thomas & Harden, 2008). It was done in four stages (Figure 1).

### Stage one: data extraction

A structured template was used to extract data from 23 studies. Study details (title, author, year, country), study methods (design, data collection methods, data analysis), and participant characteristics (recruitment setting, stakeholder type and sample size, age and gender) were documented. One reviewer (D.A.) extracted data pertaining to the behaviors of interest including all barriers, facilitators, and factors influencing hearing and vision care in LTC. For qualitative studies, relevant quotations and verbatim findings were extracted. For quantitative studies, survey responses, supporting data and authors’ narrative descriptions of findings were extracted. Another reviewer (C.M.) checked this extraction process. All relevant findings were extracted regardless of statistical significance or reporting frequency, as behavioral systems mapping prioritizes contextual completeness of data for the mapping process.

### Stage two: free-text coding

Data were then coded based on meaning and context, with free text code added to the codebook by reviewer (D.A.). New codes were created as needed. Following this, the actors and behaviors associated with each were identified based on the context and implications. A second reviewer (C.M.) experienced in behavioral systems mapping and COM-B application was actively involved throughout the coding process through regular collaborative meetings with D.A. reviewer. Both reviewers had a clear understanding of coding objectives and COM-B framework application. Any discrepancies were resolved through iterative discussion and referring to the original articles until consensus was achieved.

For example*, “the majority of managers (78%) reported that their facility did not provide in-house hearing testing” (Page number: 1522*, Leroi et al., 2021*)* Code*: Lack of onsite or nearby hearing service |* Actor: *LTC* | Behavior: *Receiving Sensory Care*

*“I leave notes and I tell staff, and if I’m there and they’re speaking to her, and I can tell Mum’s not picking up the conversation, I just say, “You’ll have to speak a bit louder,” or “Come closer” (Family member)” (Page number: 852, Bott et al., 2022)* Code: *Family member provide communication technique |* Actor: *Family member* | Behavior: *Communicating with residents*

### Stage three: grouping factors and linking to actors, behaviors and COM-B

The codes in the codebook were reviewed and grouped into factors by two reviewers (D.A. and C.M.). Any conflicts were resolved by a third reviewer from the team. Each of these factors were labelled to denote neutrality in the variables in the behavioral systems map. For example, “awareness of tools/protocols”, rather than *increased or decreased* awareness of tools/protocols. The factors were then linked to COM-B components (i.e., physical or psychological capability, social or physical opportunity, or reflective or automatic motivation), along with the corresponding behaviors they influenced and the actors responsible for each factor. The primary research team (D.A., C.M., P.D., L.K., S.K.) who were subject matter experts (audiology, optometry, aged care research, and behavioral science) then reviewed all factors and their corresponding codes (Appendix 5—see online supplementary material) to ensure factors were labelled appropriately and accurately categorized into COM-B components. Any overlapping factors were collapsed to ensure each factor was distinct.

### Stage four: behavioral systems mapping

**Figure 2:**
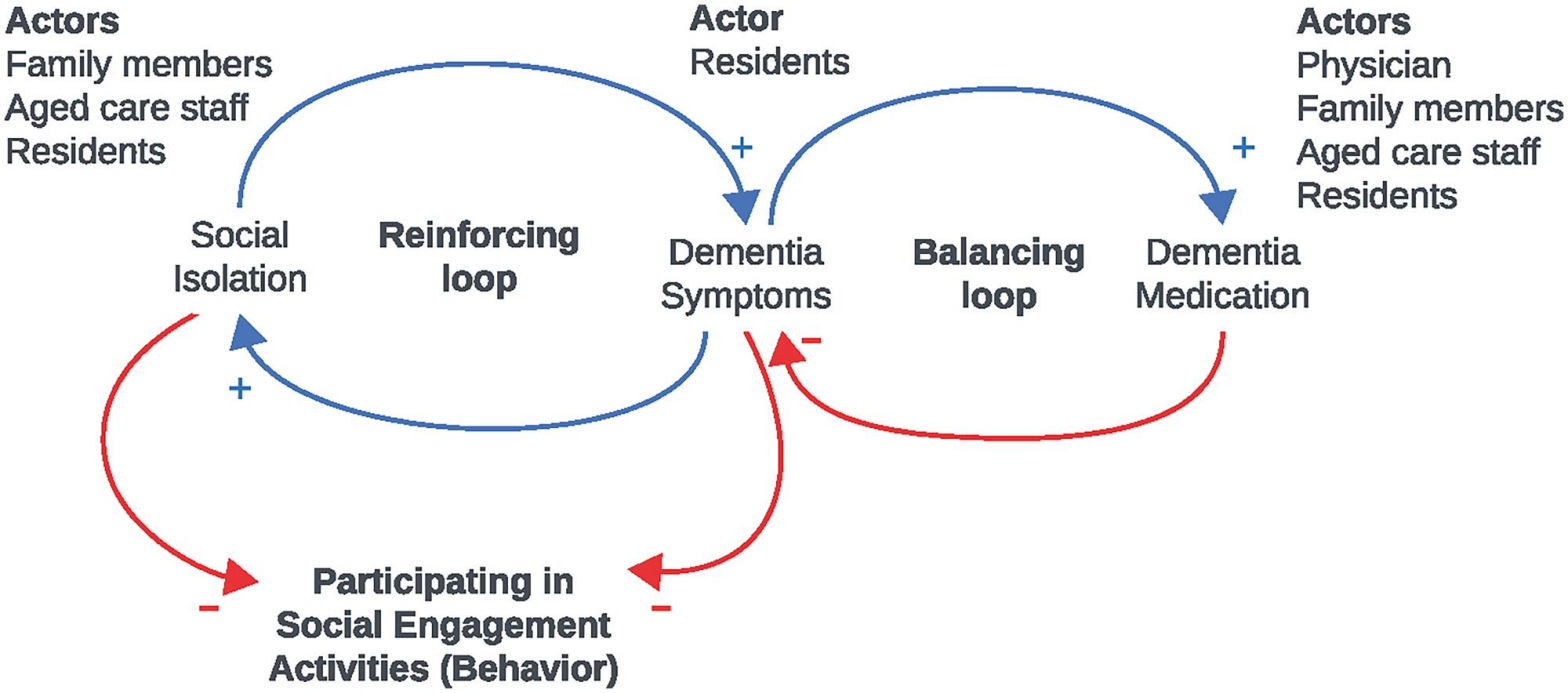
Example of reinforcing and balancing feedback loops influencing participation in social engagement activities (behavior) among residents in LTC (long-term care).

BSM comprises three key elements: actors (represented by labels), behaviors, factors influencing behaviors (expressed as variables), and causal relationships or influences (represented as arrows). These connections are represented as directed arrows between variables, indicating that the first variable is theorized to mechanistically affect the second variable in a probabilistic way (Hale et al., 2022; West, 2020). Each connection was marked as ‘positive’ (same direction of change; as X increases, Y also increases and vice versa) or ‘negative’ (opposite direction of change; as X increases, Y decreases and vice versa). For instance, see Figure 2, behavior of participating in social engagement activities in LTC. When social isolation increases, the risk and severity of dementia symptoms also increase. As dementia symptoms worsen, residents further withdraw socially, leading to increased isolation, forming a reinforcing loop. On the other hand, with dementia symptoms, the use of dementia medication increases; effective medication can reduce dementia symptoms, forming a balancing loop. Both social isolation and dementia negatively influence residents’ participation in social engagement activities, whereas dementia medication influences this behavior by moderating symptoms.

Initially, Inter-Relationship diagrams (IRD) for each behavior were developed (Appendix 6—see online supplementary material) and researchers collaborated to create the behavioral systems map: two vision experts (L.K. and S.K.) and two hearing experts (P.D. and C.M.) in geriatric and aged care, and a doctoral candidate with a vision science background (D.A.). We utilized the set of factors (variables) from stage 3 to establish meaningful connections between variables that influence the target behaviors. To ensure that logical connections between the factors were established, each behavior was introduced systematically, starting with screening. As we had also identified common factors across the behaviors, we drew meaningful links either with the behavior or factors. The behavioral systems map was reviewed and refined iteratively throughout the mapping process. Finally, the key loops of the system were identified and labelled. The loops could either be reinforcing or balancing the behavioral outcome. Reinforcing feedback loops create an amplifying effect, where an initial change in a variable triggers additional change in that same variable and direction. Conversely, in balancing feedback loops, changes in a variable prompt counteractive responses that balance or stabilize the system. The behavioral systems map was drawn using Kumu.io (https://kumu.io), a web-based relationship mapping platform based in the United States of America. The completed map was reviewed by a systems practitioner (S.M.) who provided guidance on the mapping structure, logical consistency of relationships, interpretation of feedback loops, and overall systems coherence.

## Results

After removing duplicates, 3,644 studies were identified. Of these, 3,528 were excluded in title and abstract screening, and 93 were excluded after full-text review. Finally, 23 articles were included in this review (Refer to online supplementary material Appendix 3 for PRISMA flow chart).

### Characteristics of the included studies and their participants

A summary of the included studies is in online supplementary material Appendix 4. The majority of studies were conducted in the UK (*n* = 6) (Andrusjak et al., 2021; Cross et al., 2023a, 2023b, 2025; Leroi et al., 2021; Pryce & Gooberman-Hill, 2013), followed by Canada (*n* = 4) (Höbler et al., 2018; Kergoat et al., 2014; Slaughter et al., 2014; Wittich et al., 2018). Two studies were conducted in South Africa (de Andrade et al., 2022; Moroe & Vazzana, 2019), India (Marmamula, et al., 2023a, 2023b), and Australia (Bott et al., 2022; Wittorff et al., 2023). One study was conducted in each of Germany (Schröder et al., 2020), Norway (Solheim et al., 2016), Scotland (White et al., 2021), South Korea (Kwak et al., 2022), Iran (Yekta et al., 2019), and Denmark (Jensen & Tubæk, 2017). One study included data from England, South Korea, India, Greece, Indonesia, and Australia (Dawes et al., 2021).

The number of LTCs represented across studies ranged from a single facility to 486. Studies with the largest number of participating LTC came from Germany (486 LTC) (Schröder et al., 2020), South Korea (286 LTC) (Kwak et al., 2022), and Canada (196 LTC) (Kergoat et al., 2014), followed by Scotland (154 LTC) (White et al., 2021), Britain (117 LTC) (Leroi et al., 2021), and India (41 LTCs) (Marmamula et al., 2023a, 2023b). In other countries, representation was limited to seven or fewer facilities, or data were not reported. Across studies, 13 used quantitative methods, seven used qualitative methods, and three employed a mixed-methods approach. Twelve studies utilized surveys for data collection (Andrusjak et al., 2021; Cross et al., 2023a, 2025; Dawes et al., 2021; Jensen & Tubæk, 2017; Kergoat et al., 2014; Kwak et al., 2022; Leroi et al., 2021; Pryce & Gooberman-Hill, 2013; Schröder et al., 2020; Solheim et al., 2016; White et al., 2021). These surveys were developed by researchers with minimal or no consultation with stakeholders, and they provided limited information on validity and reliability. All survey studies that included aged care staff as participants used online data collection methods except for one that used a postal survey (Schröder et al., 2020) and another that used in-person, online, and postal surveys (Dawes et al., 2021). Face-to-face data collection methods were used to survey residents. All qualitative studies employed in-depth interviews as their data collection method. The mixed-methods studies utilized both surveys and in-depth interviews.

Various stakeholder groups were represented in the existing studies. Aged care staff were included in 16 studies (Andrusjak et al., 2021; Bott et al., 2022; Cross et al., 2023a, 2023b; Dawes et al., 2021; Höbler et al., 2018; Jensen & Tubæk, 2017; Kergoat et al., 2014; Kwak et al., 2022; Leroi et al., 2021; Pryce & Gooberman-Hill, 2013; Schröder et al., 2020; Slaughter et al., 2014; Solheim et al., 2016; White et al., 2021; Wittorff et al., 2023). Aged care staff included one or more professional groups, such as nurses, allied health professionals, domestic assistants, and managers. Residents were included in six studies (Bott et al., 2022; de Andrade et al., 2022; Marmamula et al., 2023a, 2023b; Moroe & Vazzana, 2019; Yekta et al., 2019). One study each involved family members (Cross et al., 2023a) and hearing and vision care professionals (Wittich et al., 2018), while one study incorporated the perspectives of all stakeholder groups (Bott et al., 2022). The sample size of quantitative studies ranged from 131 to 1182, with a median of 400. The number of participants in qualitative studies were between 8 and 24. Among mixed-method studies, 6–12 participants were interviewed, and 12–87 were surveyed. The data extracted from the quantitative studies were descriptive rather than inferential analyses. The analysis methods in qualitative studies were predominantly thematic analysis, while one study each utilized grounded theory and interpretive description.

### ABC (actors–behaviors–COM-B factors) of hearing and vision care pathway

#### Actors

We identified five key actors contributing to the hearing and vision care pathway in LTC: residents, their family members, aged care staff, LTC management, and hearing and vision care professionals such as audiologists and optometrists.

#### Behaviors

We found that behaviors aligned with four of five previously described behaviors: screening, referring, receiving care, and device use. A fifth behavior, “communicating to residents with hearing or vision loss” was renamed to “adapting communication” because data in the literature related to factors influencing modification of communication techniques rather than “communicating” per se. Additionally, a sub-behavior, “adaptation of techniques,” was identified. A sub-behavior is an action or procedure that functions within a larger behavior (Michie et al., 2013). In this context, “adaptation of techniques” is a sub-behavior of the “screening” and “receiving” behaviors.

#### COM-B factors

A total of 31 factors influenced hearing and vision care pathways, either directly or indirectly. These 31 factors were comprised of two factors related to physical capability, five to psychological capability, 11 to physical opportunity, three to social opportunity, eight to reflective motivation, and two to automatic motivation.

#### The ABC: the actors–behaviors–COM-B factors

Overall, “use of device” behavior was influenced by the 20 factors, “screening” by 15 factors, “receiving” by 12, “adapting communication” by ten, and “referring” by six factors. In terms of actors, aged care staff played a role in 21 factors, while residents were involved in 17 factors. Family members and the LTC management were associated with nine factors each, and hearing and vision professionals were associated with seven. Refer to Table 1 for the charting of COM-B factors, actors, and behaviors, online supplementary material Appendix 5 for the table of quotes/verbatim data extraction for each factor and its codes, and online supplementary material Appendix 6 for the Inter-Relationship Diagram for each behavior.

**Table 1:** Charting of COM-B factors, actors, and behaviors.

Factors	Study reference	COM-B domain	Actors	Behavior
**Ability to pay**	(Cross et al., 2023a, 2025; Dawes et al., 2021; Kwak et al., 2022; Marmamula et al., 2023b)	Physical opportunity	Residents	Receiving | Use of device
**Adaptation of techniques (sub-behavior)**	(Cross et al., 2023b; Höbler et al., 2018; Wittich et al., 2018)	–	Professionals | staff	Screening | Receiving
**Adapting communication (sub-behavior)**	(Bott et al., 2022; Höbler et al., 2018; Pryce & Gooberman-Hill, 2013; Slaughter et al., 2014)	–	Family members | Residents | Staff	Adapting communication
**Awareness of resident’s sensory status**	(Dawes et al., 2021; Jensen & Tubæk, 2017; Kergoat et al., 2015; Leroi et al., 2021; Pryce & Gooberman-Hill, 2013; Slaughter et al., 2014; Wittorff et al., 2023)	Psychological capability	Staff	Screening | Use of device
**Awareness of tools and pathways**	(Andrusjak et al., 2021; Dawes et al., 2021; Leroi et al., 2021; Pryce & Gooberman-Hill, 2013)	Psychological capability	Staff	Screening | Referring | Use of device
**Cognitive impairment**	(Bott et al., 2022; Cross et al., 2023a, 2023b, 2025; Höbler et al., 2018; Slaughter et al., 2014; Wittorff et al., 2023)	Psychological capability	Family members | Residents | Staff	Screening | Referring | Use of device
**Collaborative care**	(Cross et al., 2025, 2023b; Höbler et al., 2018; Leroi et al., 2021; Wittich et al., 2018)	Social opportunity	Family members | Professionals | Residents | Staff	Screening | Referring | Receiving | Use of device | Adapting communication
**Communication**	(Wittich et al., 2018)	Psychological capability	Residents	Screening
**Confidence**	(Andrusjak et al., 2021; Cross et al., 2023b; Dawes et al., 2021; Kwak et al., 2022; Leroi et al., 2021; Pryce & Gooberman-Hill, 2013)	Reflective motivation	Staff	Screening | Use of device
**Device comfort**	(Cross et al., 2024, 2023a; Dawes et al., 2021; Kwak et al., 2022; Leroi et al., 2021; Marmamula et al., 2023a; Moroe & Vazzana, 2019; Solheim et al., 2016)	Automatic motivation	Residents	Use of device
**Device management**	(Andrusjak et al., 2021; Cross et al., 2024, 2023a; de Andrade et al., 2022; Moroe & Vazzana, 2019; Solheim et al., 2016; White et al., 2021)	Physical capability	Residents | Staff	Use of device
**Emotional distress**	(Cross et al., 2023a, 2023b; de Andrade et al., 2022)	Automatic motivation	Family members | Residents | Staff	Receiving | Adapting communication
**Family engagement**	(Bott et al., 2022; Cross et al., 2023a, 2025; Kwak et al., 2022; Marmamula et al., 2023b; Pryce & Gooberman-Hill, 2013; Wittich et al., 2018)	Social opportunity	Family members	Screening | Referring | Receiving | Use of device | Adapting communication
**Fellow residents’ support**	(de Andrade et al., 2022)	Social opportunity	Residents	Adapting communication
**Functional device availability**	(Cross et al., 2023a, 2025; Dawes et al., 2021; Kwak et al., 2022; Leroi et al., 2021; White et al., 2021; Yekta et al., 2019)	Physical opportunity	LTC management | Professionals | Residents | Staff	Use of device
**Infection control**	(Cross et al., 2025)	Physical opportunity	LTC management | Residents | Staff	Receiving | Adapting communication
**Infrastructure**	(Andrusjak et al., 2021; Bott et al., 2022; Cross et al., 2023b, 2025; Kergoat et al., 2014; Wittich et al., 2018)	Physical opportunity	LTC management | Professionals | Residents | Staff	Screening | Receiving | Adapting communication
**Knowledge**	(Bott et al., 2022; Cross et al., 2023a, 2023b, 2025; Höbler et al., 2018; Pryce & Gooberman-Hill, 2013; Slaughter et al., 2014; Solheim et al., 2016; White et al., 2021; Wittorff et al., 2023)	Psychological capability	Family members | Residents | Staff	Screening | Use of device
**Logistics**	(Cross et al., 2023b; Pryce & Gooberman-Hill, 2013)	Physical opportunity	LTC management | Professionals | Staff	Receiving
**Meeting expectations**	(de Andrade et al., 2022; Moroe & Vazzana, 2019; Solheim et al., 2016)	Reflective motivation	Professionals | Residents |	Use of device| adapting communication
**Mobility**	(Marmamula et al., 2023b)	Physical capability	Residents	Receiving
**Onsite service**	(Andrusjak et al., 2021; Cross et al., 2023b, 2025; Höbler et al., 2018; Kergoat et al., 2014; Leroi et al., 2021; Schröder et al., 2020; White et al., 2021)	Physical opportunity	LTC management | Professionals	Receiving
**Onus of responsibility**	(Cross et al., 2023a, 2023b, 2025; Pryce & Gooberman-Hill, 2013)	Reflective motivation	Family Members | Staff	Referring | Use of device| Adapting communication
**Perception of values**	(Bott et al., 2022; Cross et al., 2023a, 2023b, 2025; Dawes et al., 2021; de Andrade et al., 2022; Höbler et al., 2018; Kergoat et al., 2014; Kwak et al., 2022; Leroi et al., 2021; Marmamula et al., 2023a; Pryce & Gooberman-Hill, 2013; Solheim et al., 2016; Yekta et al., 2019)	Reflective motivation	Family members | Professionals | Residents | Staff	Screening | Receiving | Use of device| Adapting communication
**Prioritization**	(Bott et al., 2022; Cross et al., 2023a, 2025; Wittich et al., 2018)	Reflective motivation	Staff	Screening | Use of device
**Protocols**	(Andrusjak et al., 2021; Cross et al., 2023b, 2025; Dawes et al., 2021; Höbler et al., 2018; Kergoat et al., 2014; Kwak et al., 2022; Leroi et al., 2021; White et al., 2021; Wittich et al., 2018; Wittorff et al., 2023)	Physical opportunity	LTC management | Staff	Screening | Referring | Use of device
**Staff engagement**	(Wittich et al., 2018)	Reflective motivation	Staff	Screening
**Time commitment**	(Cross et al., 2023b; Pryce & Gooberman-Hill, 2013; Wittorff et al., 2023)	Physical opportunity	LTC management | Staff	Receiving | Use of device
**Training**	(Bott et al., 2022; Cross et al., 2023a, 2023b, 2025; Dawes et al., 2021; Höbler et al., 2018; Leroi et al., 2021; Moroe & Vazzana, 2019; Pryce & Gooberman-Hill, 2013; Solheim et al., 2016; White et al., 2021; Wittorff et al., 2023)	Physical opportunity	Family members | LTC management | Residents | Staff	Screening | Use of device | Adapting communication
**Transportation**	(Cross et al., 2025; Kergoat et al., 2014; Pryce & Gooberman-Hill, 2013)	Physical opportunity	LTC management	Receiving
**Value of care plan**	(Dawes et al., 2021; Wittorff et al., 2023)	Reflective motivation	Staff	Use of device
**Willingness**	(Andrusjak et al., 2021; Bott et al., 2022; Cross et al., 2023a, 2023b, 2025; Pryce & Gooberman-Hill, 2013)	Reflective motivation	Residents	Use of device
**Workforce stability**	(Slaughter et al., 2014)	Physical opportunity	Staff	Screening

*Note*. LTC: Long-term care; Staff refers to registered nurses, enrolled nurses, care staff, personal care workers, and other direct care personnel; Professionals refer to hearing professionals (audiologist or ear, nose, and throat (ENT) specialist), and vision professionals (optometrist or ophthalmologist); Device refers to hearing aids, cochlear implants, spectacles, magnifying glasses, low vision aids, or assistive software.

Eighteen factors were common influencing factors across two or more behaviors. The rest of the factors were specific to a particular behavior. Factors such as “collaborative care” and “family engagement” emerged as influential factors across all five behaviors. Additionally, “Infrastructure” in LTCs and “perception of values” factors also influenced all behaviors except for “referring” behavior.

#### Behavioral systems mapping

**Figure 3:**
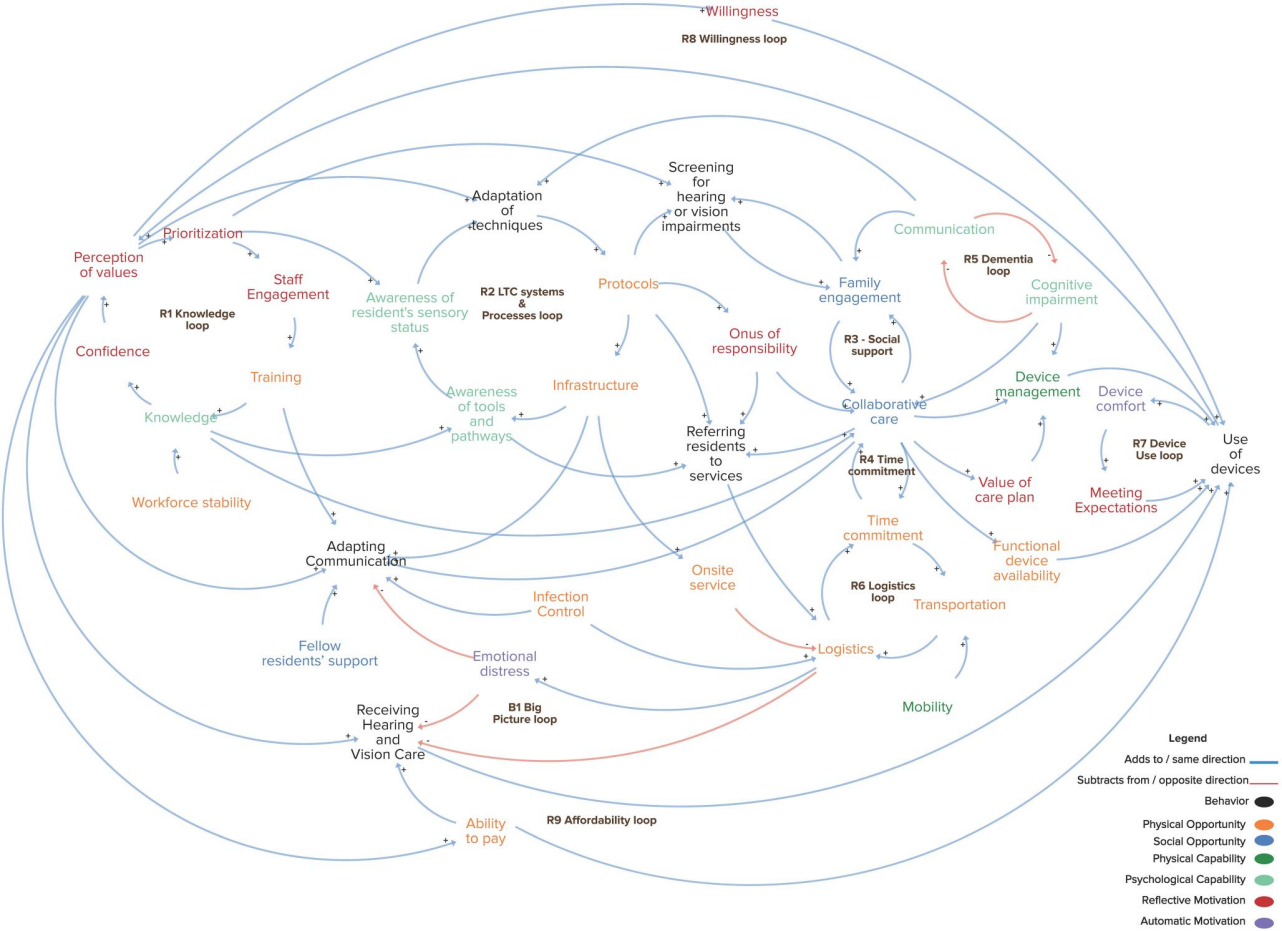
Behavioral systems map showcasing all nine reinforcing loops and one balancing loop.

Nine reinforcing and one balancing feedback loops were identified as potential drivers of behaviors (see Table 2). In every loop, one or more actors shaped the factors and influenced behavior. Figure 3 illustrates the static layout of the map. Click this link, Behavioral Systems Map of ABC to access the interactive map. The map interface shows factors color-coded by COM-B components, with connection types indicated by directed arrows (blue arrows = same direction relationships; red arrows = opposite direction relationships). Interactive toggle features at the bottom of the map allow filtering the map by: COM-B components, Actors, and Behaviors. Clicking individual factors displays a navigation pane showing supporting quotations from source articles, associated actors, and influenced behaviors. Clicking on loop labels in the map, then clicking the focus icon (⊕) in the right highlights and isolating each loop pathway. Feedback loops R1 through R9 represent reinforcing cycles. These cycles can either facilitate or impede hearing and vision care behaviors in LTC facilities, depending on whether they operate as beneficial direction or detrimental direction cycles within the system. These feedback loops are explained in positive terms below; if the factors acted negatively (decreasingly), it would weaken the cycle, and lead to poor sensory care behaviors. The B1 'Big Picture’ loop acts as a balancing feedback loop, shaping behaviors from screening to referring residents to services to receiving care to device use.

**Table 2:** Feedback loops and its explanation.

			Actors in the loop (showing interdependent roles operating in parallel across feedback loops)				
Behavior	Feedback loops	Explanation	Family member	Resident	Aged care staff	Long term care management	Hearing/vision professionals
**Screening for hearing or vision impairments**	**R1 Knowledge:** training—knowledge—confidence—perception of value—prioritization—staff engagement—training	Increased training would enhance knowledge in sensory screening and management of sensory impairment. This could potentially lead to more confidence and increase the perception of values of sensory care. The latter might lead to more prioritization and, thus, the engagement of staff with the residents; thereby, they seek more training, forming a closed loop.	Training—knowledge—perception of value	Training—knowledge—perception of value	Training—knowledge—confidence—perception of value—prioritization—staff engagement—training	Training	Perception of value
**Screening for hearing or vision impairments**	**R2 LTC systems and processes:** protocol—infrastructure—awareness of tools and pathways—awareness of residents’ sensory status—adaptation of techniques—protocol	Having a protocol for sensory care support in LTC might facilitate provision to appropriate infrastructure, including environmental settings, tools, and equipment. This, in turn, would increase staff awareness of available resources and protocols in place. This might enable awareness of residents’ sensory status and lead to adaptation of screening techniques to suit residents’ needs. Finally, the loop is closed by informing the development of improved LTC protocols that further strengthen sensory care.	–	–	Protocol—infrastructure—awareness of tools and pathways –awareness of residents’ sensory status—adaptation of techniques—protocol	Protocol—infrastructure	Infrastructure—adaptation of techniques
**Screening for hearing or vision impairments** **Referring residents to services** **Receiving hearing and vision care** **Use of devices** **Adapting communication to sensory loss**	**R3 Social support**: family engagement—collaborative care—family engagement	Increased family engagement in a resident’s care may foster collaborative care and support, which in turn would require more involvement from family members.	Family engagement— collaborative care	Collaborative care	Collaborative care	–	Collaborative care
**Receiving hearing and vision care** **Use of devices**	**R4 Time commitment:** collaborative care—time commitment—collaborative care	When collaborative care improves, staff may have more time to dedicate to care, and greater time availability, in turn, reinforces collaborative care	Collaborative care	Collaborative care	Collaborative care— time commitment	Time commitment	Collaborative care
**Screening for hearing or vision impairments**	**R5 Dementia:** cognitive impairment—communication—cognitive impairment	As cognitive impairment worsens, communication difficulties increase, which may exacerbate the impacts of cognitive impairment or dementia	Cognitive impairment	Cognitive impairment— communication	Cognitive impairment	–	–
**Receiving hearing and vision care**	**R6 Logistics:** time commitment—transportation—logistics—time commitment	As time commitment towards residents increases, it enables better support with transportation needs. However, this enhanced support often necessitates multiple visits and creates logistic challenges, which in turn require even more time commitment from staff, thus reinforcing the cycle.	–	–	Time commitment—logistics	Time commitment—transportation—logistics	Logistics
**Use of devices**	**R7 Device use:** use of device—device comfort—meeting expectations—use of device	Using the device allows residents to assess its comfort (fit and quality of hearing/vision). As comfort improves, it would meet expectations, promoting device use and thus continuing the cycle.		Use of device— device comfort—meeting expectations			Meeting expectations— use of devices
**Use of devices**	**R8 Willingness:** perception of values- willingness—use of devices—perception of values	As perception of values increases, willingness to use devices increases, which becomes self-reinforcing.	Perception of value	Perception of value— willingness— use of devices	Perception of value		Perception of value
**Use of devices**	**R9 Affordability:** perception of values-ability to pay-use of device—perception of values	As perception of values increases (through professional recommendations, peer experiences, or observed benefits), individuals pay to buy devices, leading to use of device, which provides firsthand experience of benefits, further reinforcing the perception of values and continuing the cycle.	Perception of value	Perception of value— ability to pay— use of devices	Perception of value		Perception of value
**Screening for hearing or vision impairments** **Referring residents to services** **Receiving hearing and vision care** **Use of Devices**	**B1 Big Picture:** knowledge—awareness of screening tools and pathways—awareness of residents’ sensory status—adaption of techniques—protocols—*screening for hearing or vision impairments* (behavior)—family engagement—collaborative care—*referring residents to sensory services* (behavior)—logistics—emotional distress—*receiving care* (behavior)—use of device—perception of value—prioritization—staff engagement—training—knowledge	As knowledge increases, awareness of screening tools and residents’ sensory status grows. This drives increased adaption of screening techniques, informing improved LTC protocols and leading to more screening behavior. This heightened screening increases the need for family engagement, fostering collaborative care and encouraging referrals to sensory services. However, these referrals trigger the need for logistics arrangements. A higher logistical burden may lead to emotional distress such as frustration, reducing receiving (care-seeking) behavior. If care is not sought from hearing and vision professionals, residents may not be appropriately managed with devices; hence, device utilization behavior will reduce. This gives a lesser perception of value, reducing prioritization of sensory needs and lowering the level of staff engagement. This creates less demand for further training, thereby negatively impacting knowledge acquisition and closing the balancing loop.	Knowledge family engagement – collaborative care—*referring residents to sensory services* (behavior)—emotional distress—*receiving care* (behavior)—use of device—perception of value—training	Knowledge—collaborative care—*referring residents to sensory services* (behavior) -emotional distress—*receiving care* (behavior) - use of device—perception of value– training	Knowledge— awareness of screening tools and pathways – awareness of residents’ sensory status – adaption of techniques – *screening for hearing or vision impairments-* collaborative care—*referring residents to sensory services* (behavior) -logistics –emotional distress—*receiving care* (behavior) - use of device—perception of value—prioritization—staff engagement—training	Logistics—training	Adaption of techniques—*screening for hearing or vision impairments-* collaborative care—*referring residents to sensory services* (behavior)— logistics— use of device—perception of value

*Note*. LTC: Long-term care; R No.: Reinforcing loop number; B No.: Balancing loop number; Device refers to hearing aids, cochlear implants, spectacles, magnifying glasses, low vision aids, or assistive software.

All five behaviors had one or the other feedback loops influencing them along with the additional factors as illustrated in the map. The screening behavior was dependent upon five feedback loops, including R1, R2, R3, R5, B1, and one factor–workforce stability. The referral behavior comprised R3, B1 and four factors namely, protocols, awareness of tools and pathways, cognitive impairment and onus of responsibility. The receiving behavior was influenced by the R3, R4, R6, B1 along with eight factors—mobility, infection control, onsite service, infrastructure, ability to pay, emotional distress, perception of values and adaptation of techniques. The use of device behavior was driven by six feedback loops, including R3, R4, R7, R8, R9, B1 and twelve additional factors–training, knowledge, confidence, prioritization, awareness of resident’s sensory status, awareness of tools and pathways, protocols, onus of responsibility, value of care plan, functional device availability, device management, and cognitive impairment. Adapting communication to sensory loss behavior was dependent on one feedback loop R3 and seven more factors–emotional distress, perception of value, training, infrastructure, onus of responsibility, fellow residents’ support, and infection control (such as facemask wear).

## Discussion and implications

This review presents a novel behavioral systems map for hearing and vision care in LTC based on actors and associated behavioral factors identified from a systematic literature review. This approach is novel because, rather than a siloed approach to tackle longstanding challenges for hearing and vision care, it adopted a holistic, system-wide perspective. The behavioral systems map shows the interdependencies between 31 factors attributed to five stakeholder groups reported to carry out hearing and vision care. Further, this review highlights the various stakeholder groups and their influence throughout the care pathway from screening to identifying sensory needs, referring for consultation with hearing and vision professionals, receiving care, using hearing and vision devices, and adopting communication strategies. The review also provides insight into the capability, opportunity and motivation factors that drive the hearing and vision care behaviors in LTC.

### Behavioral systems mapping

BSM provides insight into the leverage points influencing behaviors that are often overlooked in conventional approaches, such as the reinforcing feedback loops that sustain or perpetuate negative behaviors, and the emotional barriers associated with receiving hearing and vision care.

Traditionally interventions focus on one groups and fail to account for systemic interdependencies (Michie et al., 2011), limiting their effectiveness in addressing the interplay of factors and behavioral determinants identified in our analysis. Previous research suggested co-developing hearing interventions with staff and residents using the Behaviour Change Wheel, a behavioral science framework (Cross et al., 2022), which aligns with our findings on the need for approaches that address behavioral components across the system.

The BSM suggests the presence of reinforcing behavioral feedback loops in the model. These loops, unchecked, could potentially accelerate unfavorable behavioral outcomes if the influencing factors act negatively. For example, consider the use of device behavior loop: if perception of value of using a device is low, residents are less likely to purchase the device. Device usage then decreases, leading to a lack of first-hand experience with the benefits of devices. This further reduces the perceived value of the device, reinforcing the cycle of non-usage of devices.

Another interesting observation is the B1—big picture balancing feedback loop, which allows us to visualize the pathway from screening to device use. This loop highlights that even if multiple factors positively contribute to screening and referring, if there is a logistical burden (such as difficulties in fixing appointments), it can cause emotional distress and counteract the effort to receive care. The emotional distress experienced by residents towards receiving care could affect use of hearing and vision devices and services. This dynamic highlights why focusing on single solutions may not be sufficient (e.g., screening alone would be unlikely to boost uptake of hearing and vision devices). This demonstrates the value of the BSM approach in revealing interconnected barriers that require multi-strategy interventions. Rather than focusing on single solutions, effective interventions must simultaneously address multiple barriers.

This prototype behavioral systems map offers initial insight to the nexus of relationships and complexity within the LTC system, highlighting the need to conceptualize these elements while planning any future investigations. For example, Holloway et al. developed a protocol for a randomized controlled trial of free domiciliary vision services, and surgical treatments in LTC (Holloway et al., 2018). The results of this trial demonstrated low uptake of further intervention (only 4 of 20 underwent cataract surgery/took further management referral) (Man et al., 2020). Based on our model, we speculate that intrinsic factors, such as perceived effectiveness, willingness to use glasses or low vision aids, meeting residents’ expectations, and support from care staff could have influenced the outcomes of the intervention. Hence, understanding the dynamics between interventions and systemic factors is essential, as improving care outcomes may require tackling multiple barriers at different levels of the system simultaneously (Homer & Hirsch, 2006).

Employing behavioral science frameworks enabled a synthesis of the wide range of factors influencing hearing and visual behaviors. Multiple capability, opportunity and motivational factors were identified in all five behavior pathways. For example, when synthesizing the use of hearing and vision devices behavior, we identified thirteen factors: four related to capability, six to opportunity, and three to motivation. Cross et al., similarly, found that staff in LTCs face multiple barriers when providing hearing support to residents with dementia; training to boost capability is insufficient, and creating opportunities in terms of time and resources is also necessary to improve care (Cross et al., 2023a). Our synthesis also emphasized that hearing and vision care in LTCs requires collaboration between multiple stakeholder groups such as hearing and vision care professionals, staff, family members, and residents, alongside formalized protocols by the LTC management. This aligns with Andrusjak et al.'s scoping review, which highlighted that improving staff knowledge in identifying sensory impairments and managing assistive devices must be complemented by resident and family involvement (Andrusjak et al., 2020). While Andrusjak et al., suggested external professionals should be associated with LTCs to support care delivery (Andrusjak et al., 2020), our findings extend this by recommending that such collaborative arrangements be explicitly incorporated by LTC protocols.

### Policy-relevant insights

Systems thinking models have been increasingly used to enhance understanding of complex multi-actor systems with the potential to inform policy and practice development across multiple fields (Littlejohns et al., 2021). BSM represents a recent innovative mapping approach that incorporates determinants of human behavior, creating links between factors influencing behavior, actors and behavioral outcomes. The present study applied BSM to hearing and vision care, capturing key behavioral relationships identified in our literature informed map, and lays the groundwork for more comprehensive mapping. Our prototype behavioral systems map indicates that policy development might benefit from incorporating behavioral dimensions alongside traditional service delivery elements (French et al., 2012). Specifically, policies could (i) mandate staff training in sensory care to build psychological capability, (ii) provide protocols for LTC to routinely assess and document psychological barriers including residents’ perception of value, willingness, and emotional responses in care planning, (iii) fund education and awareness programs for aged care staff, residents and families to build knowledge and perception of sensory care value.

### Strengths, limitations and future directions

As far as we know, this is the first study to model the hearing and vision care behaviors within LTC using systems thinking methodology. With few exceptions, previous studies have not applied a behavioral science approach to systematically examine barriers and enablers to specific care behaviors, suggesting that additional influences are likely unidentified. The current map represents a rigorous synthesis of available literature but it is limited by what has been published. For instance, existing literature addresses barriers to device usage, but the operational challenges within LTC, such as protocols for handling device malfunctions, replacement of lost devices, and coordination of hearing and vision care professionals’ visits remain underexplored. The procedural gaps in facilitating hearing and vision assessments for residents, particularly when facilities lack on-site services, represent a significant yet under-researched component of the care pathway. Our concern is that these gaps in the evidence limit the completeness of the behavioral systems map, potentially obscuring key leverage points.

Our BSM was informed by findings from eligible studies across 13 different countries. While some challenges may be common, some findings may not be transferable and must be interpreted judiciously. Unfortunately, this results in a lack of contextual granularity. While the BSM captures system-level relationships, it cannot identify how these dynamics vary by country or organizational context. For example, each country could have factors, such as government policies and governing bodies, funding mechanisms, resource availability, and healthcare assistance, that influence the system (Macdonald et al., 2024; World Health Organization, 2015). Additionally, cultural and physical determinants may influence care delivery (World Health Organization, 2015). Stakeholder group representation was uneven across studies and varied across countries, with limited data available on the perspectives of certain actors such as family members, hearing and vision professionals, and policymakers. The majority of studies included the perspectives of staff (Andrusjak et al., 2021; Cross et al., 2023a, 2023b; Höbler et al., 2018; Kergoat et al., 2014; Kwak et al., 2022; Leroi et al., 2021; Pryce & Gooberman-Hill, 2013; Schröder et al., 2020; Slaughter et al., 2014; Solheim et al., 2016; Wittorff et al., 2023), while only two studies included the perspective of family members (Bott et al., 2022; Cross et al., 2025). For instance, only aged care staff perspectives were included in studies done in Germany (Schröder et al., 2020) and South Korea (Dawes et al., 2021; Kwak et al., 2022), limiting insights from other key stakeholder groups. Likewise, perspectives of residents were included in studies from Australia (Bott et al., 2022), India (Marmamula et al., 2023a, 2023b), Iran (Yekta et al., 2019) and South Africa (de Andrade et al., 2022; Moroe & Vazzana, 2019). Perspectives of hearing or vision professionals were reported only in two studies from Australia (Bott et al., 2022) and Canada (Wittich et al., 2018), despite their essential role in assessment and intervention in hearing and vision care. This uneven representation of actors may have constrained the identification of their roles in the factor synthesis and within feedback loops, and limited understanding of how system-level factors have affected these groups.

Sample overlap exists within the included studies which should be considered when interpreting overall findings. Cross et al. (2023b) recruited participants from Cross et al. (2023a) survey sample. However, each study has distinct aims and methods—online survey exploring hearing support provision (Cross et al., 2023a) versus qualitative interviews to identify barriers to improve hearing support (Cross et al., 2023b). Marmamula et al. (2023a) and Marmamula et al. (2023b)—both publications report findings from the same HOMES (Hyderabad Ocular Morbidity in Elderly Study) cohort of 1,182 residents. Marmamula et al. (2023a) report reasons for not wearing spectacles, whereas, Marmamula et al. (2023b) report barriers to uptake of referral eye care services (free) among those who were referred.

This BSM should be interpreted with an understanding that it represents subjective views of the systemic relationships (Uleman et al., 2024). The connections between factors depict influences—either direct impacts on succeeding factors or on the causal beliefs within the system which is probabilistic (Hale et al., 2022; Uleman et al., 2024). Experts in hearing, vision and aged care developed this model, while the perspectives of residents and other key stakeholder groups were not included due to resource constraints. We are currently engaging with residents, staff, policymakers, and other stakeholder groups to obtain these perspectives and co-develop a future iteration of a behavioral systems model for hearing and vision support in LTC.

A notable gap in this review was the absence of original studies covering the role of government policy in the provision of hearing and vision care in LTC. While the importance of policy is recognized in WHO reports and scholarly reviews (Anantharaman et al., 2025; Sloane et al., 2021; World Health Organization, 2015), such perspectives were not investigated in the primary studies included in this review. However, our search strategy focused on healthcare and clinical databases and did not include policy-specific databases, government repositories, or grey literature sources. Consequently, policy-level empirical research may be underrepresented in our synthesis. Future research could explore policy-related issues to strengthen the BSM and provide a more comprehensive understanding of the dynamics of hearing and vision care.

Our findings have practical implications for multiple stakeholder groups. The BSM reveals that system-level changes are essential to initiate and sustain positive feedback loops. LTC Administrators could allocate resources for staff training and awareness programs (R1), establish formal protocols and provide infrastructure for sensory care (R2), facilitate time allocation for staff to provide collaborative care (R4), and provide logistical support including transportation assistance (R6, B1). Without this system-level support, individual staff or professional efforts may remain fragmented. Aged care staff play a pivotal role in the system. They need training to build knowledge, confidence, and perception of value (R1), implement protocols and adapt screening techniques (R2), facilitate family engagement (R3), deliver collaborative care (R4, R6), and facilitate habitual use of hearing and vision devices (R8, R9). It is recommended that hearing and vision professionals collaborate with aged care staff in care coordination (R3, R4, B1), adapt service delivery by offering on-site services or telehealth options to minimize transportation barriers (R2, R6, B1), and support device success through proper fitting of hearing and vison devices and by meeting resident’s expectations (R7). Finally, both family members and residents should engage in training and awareness programs to build knowledge and perception of value of addressing hearing and vision needs (R1, R8, R9) and participate actively in care planning and collaborative care (R3, R4).

Additionally, our findings, similar to systems approaches used in other areas such as falls prevention (Wen et al., 2023), suggest that BSM could be valuable tools for examining other health services within LTC, including physiotherapy, occupational therapy, medication management, and nutritional care.

In conclusion, our synthesis of findings from a systematic review of hearing and vision care in LTC into a behavioral systems map, reveals the interplay between actors, behaviors, and COM-B factors influencing care provision. Existing research has largely examined care provision in relation to individual actors, overlooking the inherent complexity of care provision. Given the global burden of sensory impairment in LTC and the ongoing neglect of hearing and vision care in LTCs, there is a pressing need for renewed commitment and coordinated action among policymakers, healthcare organizations, and LTC providers to transform current practice. The novel BSM developed in this study offers a foundational prototype that could be adapted to reflect unique contextual factors in specific settings when planning and implementing interventions to facilitate and improve hearing and vision care. Future research should focus on empirically validating the relationships depicted in the model and evaluating the effectiveness of interventions informed by this systems perspective to ensure sustainable and impactful improvements in sensory care for LTC residents.

## Supplementary Material

gnag020_Supplementary_Data

## Data Availability

This systematic review protocol was registered with PROSPERO (registration number: CRD42023473031). This systematic review synthesized data from published primary studies. All included studies and their citations are provided in the reference list and supplementary materials. The data extraction forms and coding frameworks developed for this review are available upon reasonable request.
