# Supplementary material for: The ABCs of hearing and vision care in long-term care communities: a systematic review and behavioral systems map of Actors, Behaviors, and COM-B factors
Source: Gerontologist. 2026 Mar 14;66(5):gnag020. doi: 10.1093/geront/gnag020 (PMC13092512; doi:10.1093/geront/gnag020)
Supplement: gnag020_Supplementary_Data [file gnag020_supplementary_data.pdf]

Manuscript title: The ABCs of Hearing and Vision Care in Long-Term Care Communities: A Systematic Review and Behavioral Systems Map of Actors, Behaviors, and COM-B Factors.

Authors: Divya Anantharaman M.Phil[1], Carly Meyer PhD[1,2], Mehwish Nisar PhD[1], Sheela Kumaran PhD FAAO[3], Lisa Keay PhD FAAO[3,4], Sue McAvoy PhD[5], Piers Dawes PhD[1]

[1]University of Queensland Centre for Hearing Research (CHEAR), School of Health and Rehabilitation Sciences, The University of Queensland, Brisbane, Queensland, Australia

[2]Bolton Clarke Research Institute, Brisbane, Queensland, Australia

[3]School of Optometry and Vision Science, Faculty of Medicine and Health, UNSW Sydney, New South Wales 2052, Australia.

[4]The George Institute for Global Health, UNSW Sydney, New South Wales 2052, Australia

[5]The University of Queensland Centre for the Business and Economics of Health, The University of Queensland, Brisbane, Queensland, Australia

Supplementary Materials:

Appendix 1: Search Strategy

Appendix 2: Quality Appraisal of Literature

Appendix 3: PRISMA flowchart

Appendix 4: Study Characteristics

Appendix 5: Summary table of quotes for each factor

Appendix 6: Inter-Relationship Diagrams for each behavior

Appendix 1: Search Strategy

("hearing loss" OR “hearing” OR "persons with hearing impairments" OR “presbycusis” OR "hearing aids" OR “ear” OR “hear” OR “deaf” OR “deaf*” OR "hard of hearing" OR "hearing rehabilitation" OR “blindness” OR "vision disorders" OR “eye” OR "visual acuity" OR “eyeglasses” OR "vision loss" OR “ocular” OR “oculars” OR "ocular vision" OR "vision, ocular" OR "low vision" OR "visual impairment" OR "vision disorder" OR “spectacles” OR “glasses” OR "sensory loss" OR "dual loss" OR "dual sensory loss" OR "dual sensory impairment") AND ("Housing for the Elderly" OR "Homes for the Aged" OR "health services for the aged" OR "nursing homes" OR "residential aged care" OR "old age home*" OR home-like OR homelike OR "aged care" OR "aged care communities" OR "long term care" OR "care home" OR "care for older adults" OR "long term care" OR "care for older adults" OR "care home*").

Appendix 2: Quality Appraisal of literature

**Author, Year**	**Category of study design**	**Methodological quality criteria**	**Yes**	**No**	**Can't tell**	**Oxford Centre for Evidence-Based Medicine (OCEBM) Levels of Evidence**
Andrusjak et al. (2021)	Quantitative Descriptive	S1: Are there clear research questions?	X			Level 3
		S2: Do the collected data allow to address the research question?	X			
		4.1. Is the sampling strategy relevant to address the research question?	1			
		4.2. Is the sample representative of the target population?	1			
		4.3. Are the measurements appropriate?	1			
		4.4. Is the risk of nonresponse bias low?		1		
		4.5. Is the statistical analysis appropriate to answer the research question?	1			
Bott et al. (2022)	Qualitative	S1: Are there clear research questions?	X			Level 3
		S2: Do the collected data allow to address the research question?	X			
		1.1. Is the qualitative approach appropriate to answer the research question?	1			
		1.2. Are the qualitative data collection methods adequate to address the research question?	1			
		1.3. Are the findings adequately derived from the data?	1			
		1.4. Is the interpretation of results sufficiently substantiated by data?	1			
		1.5. Is there coherence between qualitative data sources, collection, analysis and interpretation?	1			
(Cross et al., 2023a)	Quantitative Descriptive	S1: Are there clear research questions?	X			Level 3
		S2: Do the collected data allow to address the research question?	X			
		4.1. Is the sampling strategy relevant to address the research question?	1			
		4.2. Is the sample representative of the target population?	1			
		4.3. Are the measurements appropriate?	1			

		4.4. Is the risk of nonresponse bias low?	1			
		4.5. Is the statistical analysis appropriate to answer the research question?	1			
Cross et al. (2023b)	Qualitative	S1: Are there clear research questions?	X			Level 3
		S2: Do the collected data allow to address the research question?	X			
		1.1. Is the qualitative approach appropriate to answer the research question?	1			
		1.2. Are the qualitative data collection methods adequate to address the research question?	1			
		1.3. Are the findings adequately derived from the data?	1			
		1.4. Is the interpretation of results sufficiently substantiated by data?	1			
		1.5. Is there coherence between qualitative data sources, collection, analysis and interpretation?	1			
Cross et al. (2024)	Qualitative	S1: Are there clear research questions?	X			Level 3
		S2: Do the collected data allow to address the research question?	X			
		1.1. Is the qualitative approach appropriate to answer the research question?	1			
		1.2. Are the qualitative data collection methods adequate to address the research question?	1			
		1.3. Are the findings adequately derived from the data?	1			
		1.4. Is the interpretation of results sufficiently substantiated by data?	1			
		1.5. Is there coherence between qualitative data sources, collection, analysis and interpretation?	1			
Dawes et al. (2021)	Quantitative Descriptive	S1: Are there clear research questions?	X			Level 3
		S2: Do the collected data allow to address the research question?	X			
		4.1. Is the sampling strategy relevant to address the research question?	1			
		4.2. Is the sample representative of the target population?	1			
		4.3. Are the measurements appropriate?		1		
		4.4. Is the risk of nonresponse bias low?	1			

		4.5. Is the statistical analysis appropriate to answer the research question?	1			
de Andrade et al. (2022)	Qualitative	S1: Are there clear research questions?	X			Level 3
		S2: Do the collected data allow to address the research question?	X			
		1.1. Is the qualitative approach appropriate to answer the research question?	1			
		1.2. Are the qualitative data collection methods adequate to address the research question?	1			
		1.3. Are the findings adequately derived from the data?	1			
		1.4. Is the interpretation of results sufficiently substantiated by data?	1			
		1.5. Is there coherence between qualitative data sources, collection, analysis and interpretation?	1			
Höbler et al. (2018)	Qualitative	S1: Are there clear research questions?	X			Level 3
		S2: Do the collected data allow to address the research question?	X			
		1.1. Is the qualitative approach appropriate to answer the research question?	1			
		1.2. Are the qualitative data collection methods adequate to address the research question?	1			
		1.3. Are the findings adequately derived from the data?	1			
		1.4. Is the interpretation of results sufficiently substantiated by data?	1			
		1.5. Is there coherence between qualitative data sources, collection, analysis and interpretation?	1			
Jensen and Tubæk (2017)	Quantitative Descriptive	S1: Are there clear research questions?	x			Level 4-5
		S2: Do the collected data allow to address the research question?	x			
		4.1. Is the sampling strategy relevant to address the research question?	1			
		4.2. Is the sample representative of the target population?	1			
		4.3. Are the measurements appropriate?		1		
		4.4. Is the risk of nonresponse bias low?	1			

		4.5. Is the statistical analysis appropriate to answer the research question?		1		
Kergoat et al. (2014)	Quantitative Descriptive	S1: Are there clear research questions?	X			Level 3
		S2: Do the collected data allow to address the research question?	X			
		4.1. Is the sampling strategy relevant to address the research question?	1			
		4.2. Is the sample representative of the target population?	1			
		4.3. Are the measurements appropriate?	1			
		4.4. Is the risk of nonresponse bias low?		1		
		4.5. Is the statistical analysis appropriate to answer the research question?	1			
Kwak et al. (2022)	Quantitative Descriptive	S1: Are there clear research questions?	X			Level 3
		S2: Do the collected data allow to address the research question?	X			
		4.1. Is the sampling strategy relevant to address the research question?	1			
		4.2. Is the sample representative of the target population?	1			
		4.3. Are the measurements appropriate?	1			
		4.4. Is the risk of nonresponse bias low?		1		
		4.5. Is the statistical analysis appropriate to answer the research question?	1			
Leroi et al. (2021)	Quantitative Descriptive	S1: Are there clear research questions?	X			Level 3
		S2: Do the collected data allow to address the research question?	X			
		4.1. Is the sampling strategy relevant to address the research question?	1			
		4.2. Is the sample representative of the target population?	1			
		4.3. Are the measurements appropriate?	1			
		4.4. Is the risk of nonresponse bias low?	1			
		4.5. Is the statistical analysis appropriate to answer the research question?	1			

Marmamula, Kumbham, et al. (2023)	Quantitative Descriptive	S1: Are there clear research questions?	X			Level 3
		S2: Do the collected data allow to address the research question?	X			
		4.1. Is the sampling strategy relevant to address the research question?	1			
		4.2. Is the sample representative of the target population?	1			
		4.3. Are the measurements appropriate?		1		
		4.4. Is the risk of nonresponse bias low?	1			
		4.5. Is the statistical analysis appropriate to answer the research question?	1			
(Marmamula, Bhoopalan, et al., 2023)	Quantitative Descriptive	S1: Are there clear research questions?	X			Level 3
		S2: Do the collected data allow to address the research question?	X			
		4.1. Is the sampling strategy relevant to address the research question?	1			
		4.2. Is the sample representative of the target population?	1			
		4.3. Are the measurements appropriate?		1		
		4.4. Is the risk of nonresponse bias low?	1			
		4.5. Is the statistical analysis appropriate to answer the research question?	1			
Moroe and Vazzana (2019)	Qualitative	S1: Are there clear research questions?	X			Level 3
		S2: Do the collected data allow to address the research question?	X			
		1.1. Is the qualitative approach appropriate to answer the research question?	1			
		1.2. Are the qualitative data collection methods adequate to address the research question?	1			
		1.3. Are the findings adequately derived from the data?	1			
		1.4. Is the interpretation of results sufficiently substantiated by data?	1			
		1.5. Is there coherence between qualitative data sources, collection, analysis and interpretation?	1			

Pryce and Gooberman-Hill (2013)	Mixed-Methods	S1: Are there clear research questions?	X			Level 3
		S2: Do the collected data allow to address the research question?	X			
		5.1. Is there an adequate rationale for using a mixed methods design to address the research question?	1			
		5.2. Are the different components of the study effectively integrated to answer the research question?	1			
		5.3. Are the outputs of the integration of qualitative and quantitative components adequately interpreted?	1			
		5.4. Are divergences and inconsistencies between quantitative and qualitative results adequately addressed?	1			
		5.5. Do the different components of the study adhere to the quality criteria of each tradition of the methods involved?	1			
Schröder et al. (2020)	Quantitative Descriptive	S1: Are there clear research questions?	X			Level 3
		S2: Do the collected data allow to address the research question?	X			
		4.1. Is the sampling strategy relevant to address the research question?	1			
		4.2. Is the sample representative of the target population?	1			
		4.3. Are the measurements appropriate?	1			
		4.4. Is the risk of nonresponse bias low?		1		
		4.5. Is the statistical analysis appropriate to answer the research question?	1			
Slaughter et al. (2014)	Qualitative	S1: Are there clear research questions?	X			Level 3
		S2: Do the collected data allow to address the research question?	X			
		1.1. Is the qualitative approach appropriate to answer the research question?	1			
		1.2. Are the qualitative data collection methods adequate to address the research question?	1			
		1.3. Are the findings adequately derived from the data?	1			
		1.4. Is the interpretation of results sufficiently substantiated by data?	1			

		1.5. Is there coherence between qualitative data sources, collection, analysis and interpretation?	1			
Solheim et al. (2016)	Quantitative Descriptive	S1: Are there clear research questions?	X			Level 3
		S2: Do the collected data allow to address the research question?	X			
		4.1. Is the sampling strategy relevant to address the research question?	1			
		4.2. Is the sample representative of the target population?	1			
		4.3. Are the measurements appropriate?	1			
		4.4. Is the risk of nonresponse bias low?	1			
		4.5. Is the statistical analysis appropriate to answer the research question?	1			
White et al. (2021)	Quantitative Descriptive	S1: Are there clear research questions?	X			Level 3
		S2: Do the collected data allow to address the research question?	X			
		4.1. Is the sampling strategy relevant to address the research question?	1			
		4.2. Is the sample representative of the target population?	1			
		4.3. Are the measurements appropriate?	1			
		4.4. Is the risk of nonresponse bias low?		1		
		4.5. Is the statistical analysis appropriate to answer the research question?	1			
Wittich et al. (2018)	Qualitative	S1: Are there clear research questions?	X			Level 3
		S2: Do the collected data allow to address the research question?	X			
		1.1. Is the qualitative approach appropriate to answer the research question?	1			
		1.2. Are the qualitative data collection methods adequate to address the research question?	1			
		1.3. Are the findings adequately derived from the data?	1			
		1.4. Is the interpretation of results sufficiently substantiated by data?	1			
		1.5. Is there coherence between qualitative data sources, collection, analysis and interpretation?	1			

Wittorff et al. (2023)	Qualitative	S1: Are there clear research questions?	X			Level 3
		S2: Do the collected data allow to address the research question?	X			
		1.1. Is the qualitative approach appropriate to answer the research question?	1			
		1.2. Are the qualitative data collection methods adequate to address the research question?	1			
		1.3. Are the findings adequately derived from the data?	1			
		1.4. Is the interpretation of results sufficiently substantiated by data?	1			
		1.5. Is there coherence between qualitative data sources, collection, analysis and interpretation?	1			
Yekta et al. (2019)	Quantitative Descriptive	S1: Are there clear research questions?	X			Level 3
		S2: Do the collected data allow to address the research question?	X			
		4.1. Is the sampling strategy relevant to address the research question?	1			
		4.2. Is the sample representative of the target population?	1			
		4.3. Are the measurements appropriate?		1		
		4.4. Is the risk of nonresponse bias low?	1			
		4.5. Is the statistical analysis appropriate to answer the research question?	1			

Appendix 3: PRISMA flowchart & checklist

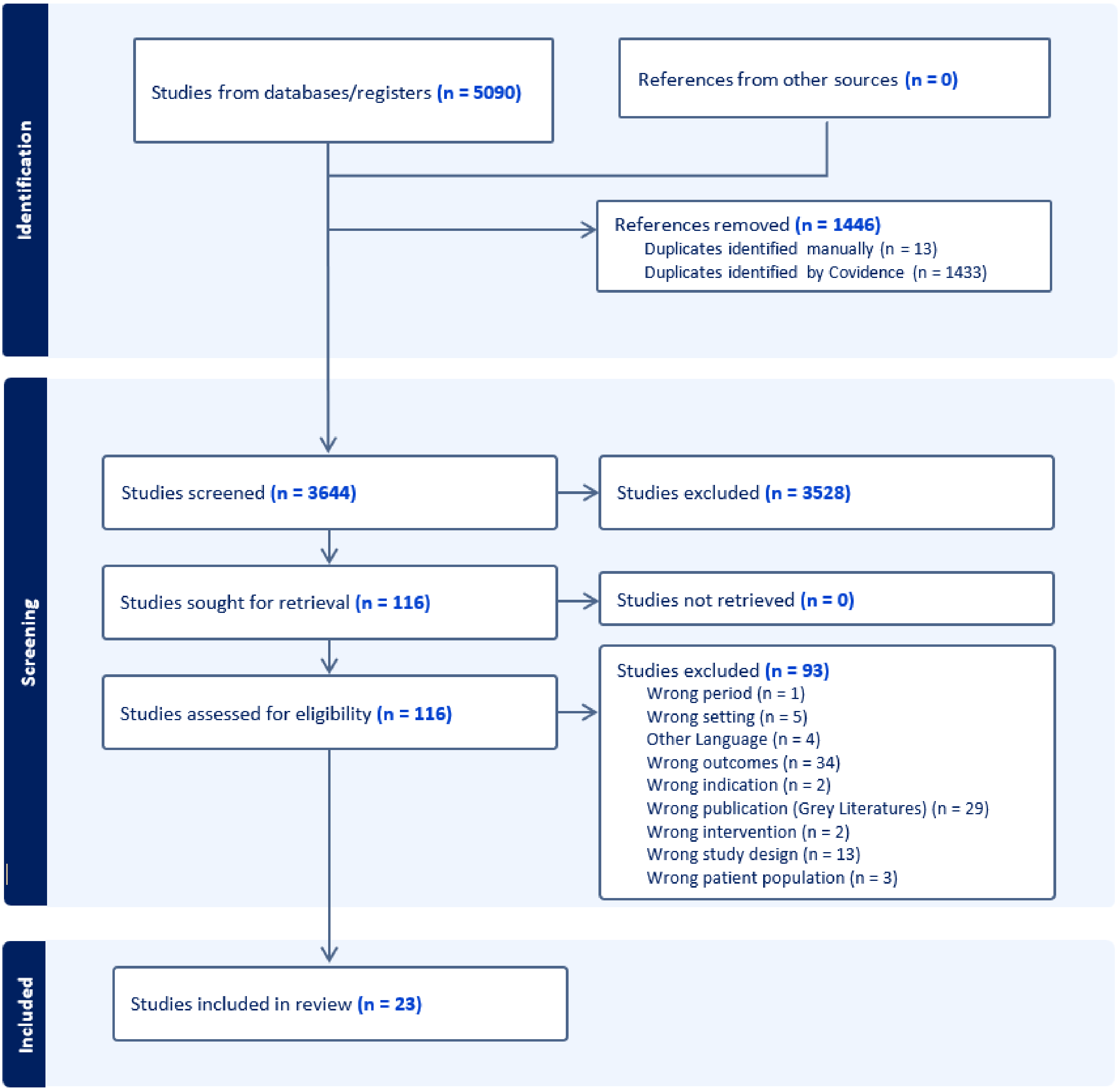

Section and Topic	Item #	Checklist item	Location where item is reported
**TITLE**			
Title	1	Identify the report as a systematic review.	Title page
**ABSTRACT**			
Abstract	2	See the PRISMA 2020 for Abstracts checklist.	Abstract
**INTRODUCTION**			
Rationale	3	Describe the rationale for the review in the context of existing knowledge.	Introduction
Objectives	4	Provide an explicit statement of the objective(s) or question(s) the review addresses.	Introduction
**METHODS**			
Eligibility criteria	5	Specify the inclusion and exclusion criteria for the review and how studies were grouped for the syntheses.	Methods - Study Selection & Quality Assessment
Information sources	6	Specify all databases, registers, websites, organisations, reference lists and other sources searched or consulted to identify studies. Specify the date when each source was last searched or consulted.	Methods – Search Strategy
Search strategy	7	Present the full search strategies for all databases, registers and websites, including any filters and limits used.	Methods – Search Strategy and Appendix 1
Selection process	8	Specify the methods used to decide whether a study met the inclusion criteria of the review, including how many reviewers screened each record and each report retrieved, whether they worked independently, and if applicable, details of automation tools used in the process.	Methods – Pg 4 & 5
Data collection process	9	Specify the methods used to collect data from reports, including how many reviewers collected data from each report, whether they worked independently, any processes for obtaining or confirming data from study investigators, and if applicable, details of automation tools used in the process.	Methods – Data Extraction and Data Synthesis Pg 4 & 5
Data items	10a	List and define all outcomes for which data were sought. Specify whether all results that were compatible with each outcome domain in each study were sought (e.g. for all measures, time points, analyses), and if not, the methods used to decide which results to collect.	Methods – Data Extraction and Data Synthesis Pg 4 & 5
	10b	List and define all other variables for which data were sought (e.g. participant and intervention characteristics, funding sources). Describe any assumptions made about any missing or unclear information.	Methods – Data Extraction and Data Synthesis Pg 4 & 5
Study risk of bias assessment	11	Specify the methods used to assess risk of bias in the included studies, including details of the tool(s) used, how many reviewers assessed each study and whether they worked independently, and if applicable, details of automation tools used in the process.	Methods - Study Selection & Quality Assessment Pg 4 and Appendix 3
Effect measures	12	Specify for each outcome the effect measure(s) (e.g. risk ratio, mean difference) used in the synthesis or presentation of results.	Not applicable
Synthesis methods	13a	Describe the processes used to decide which studies were eligible for each synthesis (e.g. tabulating the study intervention characteristics and comparing against the planned groups for each synthesis (item #5)).	Methods - Study Selection
	13b	Describe any methods required to prepare the data for presentation or synthesis, such as handling of missing summary statistics, or data conversions.	Methods - Study Data Extraction and Data Synthesis
	13c	Describe any methods used to tabulate or visually display results of individual studies and syntheses.	Methods - Study Data Extraction and Data Synthesis
	13d	Describe any methods used to synthesize results and provide a rationale for the choice(s). If meta-analysis was performed, describe the model(s), method(s) to identify the presence and extent of statistical heterogeneity, and software package(s) used.	Methods - Study Data Extraction and Data Synthesis
	13e	Describe any methods used to explore possible causes of heterogeneity among study results (e.g. subgroup analysis, meta-regression).	Not applicable
	13f	Describe any sensitivity analyses conducted to assess robustness of the synthesized results.	Not applicable
Reporting bias assessment	14	Describe any methods used to assess risk of bias due to missing results in a synthesis (arising from reporting biases).	Methods - Study Data Extraction and Data Synthesis
Certainty assessment	15	Describe any methods used to assess certainty (or confidence) in the body of evidence for an outcome.	Methods - Study Data Extraction and Data Synthesis
**RESULTS**			
Study selection	16a	Describe the results of the search and selection process, from the number of records identified in the search to the number of studies included in the review, ideally using a flow diagram.	Appendix 4
	16b	Cite studies that might appear to meet the inclusion criteria, but which were excluded, and explain why they were excluded.	Appendix 4
Study characteristics	17	Cite each included study and present its characteristics.	Appendix 5
Risk of bias in studies	18	Present assessments of risk of bias for each included study.	Appendix 2
Results of individual studies	19	For all outcomes, present, for each study: (a) summary statistics for each group (where appropriate) and (b) an effect estimate and its precision (e.g. confidence/credible interval), ideally using structured tables or plots.	Not applicable
Results of syntheses	20a	For each synthesis, briefly summarise the characteristics and risk of bias among contributing studies.	Appendix 3
	20b	Present results of all statistical syntheses conducted. If meta-analysis was done, present for each the summary estimate and its precision (e.g. confidence/credible interval) and measures of statistical heterogeneity. If comparing groups, describe the direction of the effect.	Not applicable
	20c	Present results of all investigations of possible causes of heterogeneity among study results.	Not applicable
	20d	Present results of all sensitivity analyses conducted to assess the robustness of the synthesized results.	Not applicable

Section and Topic	Item #	Checklist item	Location where item is reported
Reporting biases	21	Present assessments of risk of bias due to missing results (arising from reporting biases) for each synthesis assessed.	Not applicable
Certainty of evidence	22	Present assessments of certainty (or confidence) in the body of evidence for each outcome assessed.	Not applicable
**DISCUSSION**			
Discussion	23a	Provide a general interpretation of the results in the context of other evidence.	Behavioural Systems Mapping Pg 14 & 15
	23b	Discuss any limitations of the evidence included in the review.	Strengths, limitations and future directions Pg 16
	23c	Discuss any limitations of the review processes used.	Strengths, limitations and future directions Pg 16
	23d	Discuss implications of the results for practice, policy, and future research.	Policy- Relevant Insights Pg 15
**OTHER INFORMATION**			
Registration and protocol	24a	Provide registration information for the review, including register name and registration number, or state that the review was not registered.	Methods
	24b	Indicate where the review protocol can be accessed, or state that a protocol was not prepared.	Methods
	24c	Describe and explain any amendments to information provided at registration or in the protocol.	Methods
Support	25	Describe sources of financial or non-financial support for the review, and the role of the funders or sponsors in the review.	Funding
Competing interests	26	Declare any competing interests of review authors.	Conflict of Interest - declared
Availability of data, code and other materials	27	Report which of the following are publicly available and where they can be found: template data collection forms; data extracted from included studies; data used for all analyses; analytic code; any other materials used in the review.	Will be supplied on request

From: Page MJ, McKenzie JE, Bossuyt PM, Boutron I, Hoffmann TC, Mulrow CD, et al. The PRISMA 2020 statement: an updated guideline for reporting systematic reviews. BMJ 2021;372:n71. doi: 10.1136/bmj.n71. This work is licensed under CC BY 4.0. To view a copy of this license, visit https://creativecommons.org/licenses/by/4.0/

Appendix 4: Study Characteristics

Author (Year)	Country	Study Type/Design	Participants (type and sample size)	Mean±SD/Median (range) age in yrs; Gender (M,F)	Method/Tools	Setting/recruitment	Analysis
Andrusjak et al. (2021)	England	Cross sectional	*N*=400 (aged care staff)	Not reported	Survey	74 Care homes (nursing home/ residential home)	Descriptive statistics<br>Chi-Square analyses
Bott A et al. (2022)	Australia	Qualitative study	*N*=23<br>audiologists (*n*=5),<br>LTC staff (*n*=9),<br>individuals with dementia and hearing impairment (*n*=2),<br>family members (*n*=7).	Family Members: Age = 65 ± 26; F=7<br>Audiologists - One audiologist in 20s, three in 30s and one in 50s.<br>Staff – Not reported<br>Residents - one male and other female in 90s	Interview	Audiology clinic in Australia - audiologists (convenience sample)<br>LTC - care staff (maximum variation sample)<br>LTC - individuals with dementia and hearing impairment (convenience sample),<br>LTC - family members (convenience sample)	Inductive thematic analysis
Cross, et al. (2023a)	UK	Cross sectional	*N* = 165 (aged care staff)<br>care assistants (54.5%),<br>senior carers (18.8%),<br>registered nurses (17.0%),<br>managers (3.0%) and<br>"other" (6.1%).	Age = 38.6±8.2 years<br>Gender: Not reported	Survey	LTC serving people with dementia	Descriptive, Chi Square, ANOVA, Regression<br>Optional open-ended qualitative responses were analyzed using inductive manifest content analysis
Cross, et al. (2023b)	UK	Qualitative study	*N* = 8 (aged care staff)	Not reported	Interview	Eight LTC	Deductive coding direct summative content analysis & followed by an inductive approach using reflective thematic analysis
Cross et al., (2024)	UK	Stage 1: Cross sectional<br>Stage 2: Qualitative study	Stage 1 - *N* = 87 (care givers)<br>Stage 2 - *N* = 06 (care givers)	Stage 1: Age= 37.4 years<br>M=35, F=52<br>Stage 2: Age = 57.8 years<br>F=6	Stage 1 = Survey<br>Stage 2 = Interview	LTC	Stage 1 - Descriptive & ANOVA<br>Stage 2 - Summative content analysis followed by Thematic analysis
Dawes et al., (2021)	England, South Korea, India, Greece, Indonesia and Australia	Cross sectional	*N*=428 (aged care staff: Nurse, allied health, care worker)	Not reported	Survey	48 LTC	Descriptive, Rasch Analysis, Regression
Andrade et al. (2022)	South Africa	Qualitative study	*N*=15 (residents)	Age range 71 to 93 yrs<br>M = 5, F =10	Interview	One LTC	Thematics analysis
Höbler et al. (2018)	Canada	Qualitative study	*N*=20 (registered nurse or dementia care staff)	Age=41.78<br>M=2, F= 18	Interview	Two LTC	Thematic content approach
Jensen and Tubæk (2017)	Denmark	Cross sectional	Ahed care staff sample size - not reported<br>A questionnaire for each resident was submitted to the staff with questions regarding their assessment of the patient's visual function		Survey and eye examination	LTC	Descriptive
Kergoat et al. (2014)	Canada (Quebec)	Cross sectional	*N*=196 (aged care staff)	F>M (Not reported in numbers)	Survey	196 LTC	Descriptive
Kwak et al. (2022)	South Korea	Cross sectional	*N* = 557 (aged care staff)<br>facility manager (n=104)<br>healthcare professionals included clinicians, nurses, social welfare workers, care workers (n=453)	38.1± 12.73<br>M=64, F=493	Survey	286 LTC	The structural equation model (SEM) was applied to identify the direct and/or indirect effects between the latent variable and the relationship between the latent variable and observed variable.
Leroi et al. (2021)	England	Cross sectional	*N*=887 (aged care staff)<br>group 1- managers (n=79)<br>group 2 - nurses and allied health professionals (n=160)<br>group 3 - paid care nonprofessional care workers (n=648)	Most respondents were female, constituting more than 80% in each group (Not reported in numbers)<br>Age=not reported	Survey using self-administered online or mail-in questionnaires directed at different grades of LTC staff who work with People with dementia.	117 LTC. Within each facility, 3 respondent groups were surveyed, all of whom worked with People with dementia: Group 1: Facility managers or deputy managers; Group 2: Nurses and allied health professionals (AHPs); Group 3: Paid nonprofessional care workers	Descriptive, RASCH & Regression

Author (Year)	Country	Study Type/Design	Participants (type and sample size)	Mean±SD/Median (range) age in yrs; Gender (M,F)	Method/Tools	Setting/recruitment	Analysis
Marmamula, Kumbham, et al. (2023)	India	Prospective cohort	*N*=731 (1182 residents were examined of which 731 were referred)	Age= 75.9±9.0 years (Referred residents)<br>M=250, F = 481	At least three months after the referral, the participants were interviewed and asked about the uptake of services, and their reasons for not attending.	41 LTC	Descriptive analyses were conducted. Multiple logistic regression analysis was done to assess the predictors for the uptake of eye care services and presented as adjusted Odds Ratio with 95% CIs.
(Marmamula, Bhoopalan, et al., 2023)	India	Cross sectional	*N*= 1182 (residents)	Age = 75±8.8 years<br>M= 418, F = 764	At baseline and as a follow up, the residents were asked about their reasons for not wearing spectacles.	41 LTC	Descriptive, Logistic regression & Odds Ratio calculated
Moroe and Vazzana (2019)	South Africa	Qualitative study	*N*=10 (residents)	Age = 74-85 yrs of age<br>M=3, F=7	Interviews	One LTC	Inductive thematic analysis
Pryce and Gooberman-Hill (2013)	UK	Mixed methods	Interviews: *N*=10 (aged care staff)<br>Survey: *N*=64 (aged care staff)	Survey: age range = 25-58 yrs<br>F=52, M = 12	Interviews & survey	Three LTC	Qualitative: Grounded Theory Approach<br>Survey: Descriptive
Schröder et al. (2020)	Germany	Cross sectional	*N*=486 (LTC participated)	Age = 48±9.8 years<br>F>M	Survey	Nursing homes (Rural>Urban)	Descriptive statistics
Slaughter et al. (2014)	Canada	Mixed Methods	*N*=12 (aged care staff)	100% female	Interviews	Five LTC	Interpretive description
Solheim et al. (2016)	Oslo and Bergen (Norway)	Cross sectional	*N*=195 (aged care staff)	Age = 40 ± 12.31 years	Survey	Seven LTC	descriptive statistics (mean ± standard deviation) and numbers (percent); Chi-square tests;
White et al. (2021)	Scotland	Cross sectional	*N*=154 (aged care staff)	Not reported	Survey with Multiple Choice Questions and Open-ended Questions	154 LTC	Basic descriptive statistics and reported by themes.
Wittich et al. (2018)	Canada	Mixed methods	*N*=11 (Hearing and Vision Professionals)	Age = not reported<br>M=1, F=10	Environmental scan - Interviews	Hearing and Vision Professionals across Canada with expertise in vision, hearing, Dual Sensory Impairment or healthcare technologies were recruited	Thematics analysis
Wittorff et al. (2023)	Australia (WA)	Qualitative study	*N*=24 (aged care staff: nurse, allied health professional, domestic assistant or manager)	Age = 43 ± 14.6<br>F>M	Interviews	LTC	Thematics analysis
Yekta et al. (2019)	Mashhad, Iran	Cross sectional	*N*=131 (residents)	Age = 77.67 (55 to >85)<br>F = 68.7%	Eye examinations done (it's not reported how reasons for not using glasses were obtained)	Seven LTC	Quantitative data analysis and percentage and 95% CI were used for data description. T test, X test, and ANOVA were used.

LTC: Long-term care

Appendix 5: Table of quotes/verbatim data extraction for each factor

Factor	Name of Code - number of times it was coded	Quotes / Verbatim extraction from articles
**Ability to pay**	Free service uptake was more by residents in subsidized homes -1<br>Type of LTC funding -1<br>Financial constraints - 4	**Receiving hearing or vision care** | Free service uptake was more by residents in subsidised homes, Marmamula, Kumbham, et al. (2023) | **Use of Device** | Staff working in privately owned homes reported providing hearing support to fewer residents with dementia that they thought would benefit (Mean=49.1) than those working in local authority homes (Mean=57.5), Cross, 2024. | Resident (not using HA) "too expensive" (1.2%), Cross H et al .2023 | hearing aid was too expensive (28.70%). Kwak C et al., 2022
**Adaptation of techniques**<br><br>**(SUB- BEHAVIOUR)**	Being flexible about the resident's subjective response - 2<br>Staff adapt screening technique - 6<br>Testing at the right time or day - 2<br>Lack of flexibility in assessment and management by Hearing care professionals - 1<br>Create friendly rapport - 1	**Screening for hearing or vision impairments** | Examiners are also instructed to observe the resident in their verbal interactions, and to use a book or newspaper with varying sizes of print to evaluate the respective functions; H2N2: "It was just from experience. At least they know, they're familiar with the letters, if you put their name on it [...] they know. Like, if you [...] put the random letters they not really might engage. But [...] they know their names, they're engaged in it. They will know.", Hobler, 2018 | I remember one woman that was singing constantly, but she would stop singing when she could hear me, so that was one way of finding information. (participant 2), Wittich W et al., 2018 | **Receiving hearing or vision care** | They [audiology] don't always understand because you say then "they won't wear it [hearing aid]" or "they don't like it" and it's like "oh what do you want me to do? I've done the mould" and that's it. They've done their job and they just leave it. (Manager, 11 years in profession), Cross H, 2023; "Sometimes the assessment needs to be done over several appointments." (participant 5); improve communication - establishing a friendly rapport with the client before screening and of reassuring and encouraging them throughout the procedure (Sometimes, repetition was key), Wittich, 2018
**Adapting communication**<br><br>**(SUB- BEHAVIOUR)**	(Staff) use gestures/ body language/ demonstrate - 4<br>(Staff & family member) - Adapt speaking style - 13<br>Staff prefer communicating at appropriate situation / time - 1<br>Eye contact - 2<br>Choose better ear - 1<br>Use multiple communication strategies - 1<br>Move Close - 1<br>(Staff) used single strategy than multiple - 1	**Communication** | Always make sure you're looking at them and they're looking at you. Talking slowly, not talking behind their back, that sort of thing. Always checking their hearing aids, as well. Make sure they're working. Ask them once they're in, are they working. That sort of thing. (Registered nurse, aged 53 years)., Wittorff et al., 2023 | So, it really comes down to clear pronunciation, not yelling at them, but speaking clearly, definite eye contact, and definite body language . . . use of if needed, written word. (Care Staff); You've just go to remember to try and stand near him, closer, and see your face where he's watching what you're saying. (family member), Bott A et al.,2022 | H2N8: "If their vision and hearing is poor, maybe you can demonstrate what you need to do, right? And if they can't see, it could be just taking their hands and putting the toothbrush in their hands and showing them that it's a toothbrush, if they can understand."; Nurses' communication strategies considered the person's privacy, preferences, as well as impairments, and were individualised not only to the resident, but appropriate in their timing. H1N8: "Maybe you approach the resident in the wrong situation. Like maybe since lots of them they were incontinent, maybe something is bothering them like pain or sun-downing or they wanted to go to the washroom; so be sure before approaching the residents, you approach them on the right time." Hobler F' et al., 2018
**Aware of resident's sensory status**	Staff knows resident with HI/VI-7<br>Staff aware of severity of HI/VI-2<br>Staff unaware of resident's with VI-2<br>Staff not aware of ocular conditions-2<br>Staff not aware of supporting resident's with dual sensory impairment-1<br>Staff focused on residents using HA-1	**Screening for hearing or vision impairments** | "knowledge," managers, nurses, and care workers reported that a number of residents in their care have either hearing and/or vision impairments and the severity of these impairments is high ("agree" or "strongly agree"), exceeding 88% in managers and 83% in the other 2 groups, Leroi I et al., 2021 | Apart from Korean and Indonesian respondents, most reported knowing which residents have a hearing/vision impairment. Dawes P et al., 2021 | In 159 cases, the staff did not think that the resident had vision problems – 119 of these residents belonged to vision group 0, which is consistent with the staff's view, but in 40 cases the resident was, in fact, visually impaired or blind. This corresponds to one in every four of the 159 residents in whom staff was unaware of any vision handicap. Jensen H et al., 2017 | many LTCF respondents indicated not knowing if their residents were affected by non-operated cataracts (23.0%), glaucoma (19.9%), and ARMD (26.5%), Keargoat H et at., 2014 | Findings from Stage 3 suggest that many staff were aware that most residents had hearing difficulties, but that a proportion do not think that this is the case, Pryce H et al., 2013 | **Use of Device** | Participants were generally not aware of having supported, or whether they were currently supporting, individuals with an acquired combined vision and hearing loss, Wittorff M et al., 2023 | Awareness of hearing problems was focused on known residents who had hearing aids, Pyrce H et al.,2013
**Awareness of tools and pathways**	Staff aware of screening tests/tools - 2<br>Staff unaware to pursue a referral for a hearing aid - 1<br>Staff aware of referral pathways - 1<br>Staff uncertain about the sensory care system in LTC - 1	**Screening for hearing or vision impairments** | Among managers (group 1), more than half agreed or strongly agreed that they were aware of brief hearing and vision screening tools that could be applied in the LTC setting, Leroi, 2021 | **Referring** | All 3 groups had good awareness and were able to use appropriate referral pathways for hearing and vision care (>60% agreed or strongly agreed), Leroi, 2021 | Staff reported that they were unaware how to pursue a referral for a hearing aid, Pyrce H et al., 2013 | The majority of respondents in Korea, Australia and England reported not being aware of referral pathways, Dawes, 2021 | **Receiving Hearing/Vision Care** | For those where access to eye care was a problem, the principal reasons were: 1) the perception that residents could not cooperate, 2) the fact that there was no professional on-site or close by to provide the services, 3) the lack of awareness of the oculovisual problems of residents, and 4) the perception that the oculovisual condition could not be improved., Keargoat, 2014 | **Use of Device** | uncertainty amongst staff regarding the hearing/vision practices the care homes they worked in were actually implementing, Andrusjak et al., 2023
**Cognitive Impairment**	Dementia caused challenge in recognizing HL - 2<br>Fidgets with HA due to cognitive impairment - 1<br>Lose HA due to cognitive impairment - 1<br>People with dementia unable to manage HA - 5<br>People with dementia use HA efficiently - 1<br>Cognitive impairment related challenge - 2<br>Family member thinks communication/ hearing is affected due to cognitive impairment - 1<br>People with dementia overlooked - 2	**Screening for hearing or vision impairments** | I (care staff) must admit, I have never seen that [refer to hearing services] happen., Bott A et al, 2022 | a participant explained that "Sometimes I think they [the residents] cannot understand, may be they have hearing problem, dementia sometimes, we don't know."; "is it because he cannot hear me or is it because of the dementia? I'm not sure"; "if she [a resident] don't respond you, you know either hearing aid not working, or either Alzheimer kick in, or both.", Slaughter S, et al,, 2014 | **Hearing/Vision Referral** | I must admit, I have never seen that [refer to hearing services] happen. (Care Staff), Bott A et al., 2022 | **Hearing/ Vision Management** | The other issue she had when the dementia started was that she kept losing them. She'd hide them. Even when she'd moved into the care home... Every week we'd spend a big portion of our time trying to find her hearing aids [...] it just got so ridiculous. (Family member-2), Cross H et al., 2024, | We've had residents eating their hearing aids. That was a bit of a worry. Finding the battery after that had been chewed you think "oh no" if they swallow a battery that could obviously be quite serious. (Registered Nurse-1, 23 years in profession), Cross H at al., 2023 | You know, you find them in funny places and so, it's really challenging, [certain] behaviors. People in here that have had hearing aids usually end up not wearing them, due to their behaviors; | ...[W]e come up against the problem that they can probably still read something in large print, but not necessarily understand what it means. (Clinical manager, aged 58 years), Wittorff H et al., 2023 | Audiologists identified that hearing aid use and level of technology is influenced by the presence of dementia, highlighting that many people living with dementia were unable to manage their hearing

Factor	Name of Code - number of times it was coded	Quotes / Verbatim extraction from articles
		aids., I don't honestly think it is because of his hearing. I think it's because of his dementia . . . He might call a pen a rock or something, just something that doesn't even relate to it. But, that's to do with the dementia, not the hearing. (Family Member); care staff identified that HI is sub-optimally managed for residents with dementia: HI isn't managed for residents with dementia, HI is overlooked, care staff don't realize people with dementia isn't wearing HA when talking to them Bott A et al., 2022
**Collaborative care**	Social circle should create awareness of hearing loss -1<br>Social circle should create awareness of hearing loss - 2<br>Collaborative effort - 3<br>Having trusted attendant for clinical test - 1<br>Collaborative effort for screening - 2<br>Misunderstanding btw staff and hearing care professionals - 1<br>Poor collaboration with hearing care professionals - 5<br>Collaborative approach to sensory needs - 1<br>poor communication b/w staff members & clinicians - 2<br>Co-staff support each other with hearing device - 1	**Screening for hearing or vision impairments** | It's not always possible for me, as an audiologist, to be "everywhere and see everyone, so to have staff members also be advocates for hearing is very important and, you know, to have residents themselves be advocates for hearing." (participant 5); The main enablers of sensory screening in residents with dementia were indeed other people. The clients themselves can become facilitators and advocates, as can family members, activation workers, as well as long-term care facility staff; The preference for familiarity, such as having a person the client trusts sitting by their side during the screening., Wittich W et al., 2018 | Participants (Family members) scored M = 6.2 (SD = 2.6) on a 0–10 scale as to whether they work alongside LTCH staff to provide hearing support., Cross, 2024 | **Receiving Hearing/Vision Care** | audiologists rarely visit LTCHs compared to other healthcare professionals; Difficulties co-ordinating and facilitating audiology appointments for residents were highlighted by four participants, mostly those in senior roles. Staff believed residents to have disadvantaged access to audiology because they live in LTC, not the community; Due to fragmented collaborations between LTCHs and audiology, most participants (60%, independent of role or workplace) felt pessimistic about arranging appointments for residents.; Audiology departments don't realise how stressful it is working with people with dementia and hearing loss. It kind of makes everything ten times harder than it already is. (Care Assistant-1, 8 years in profession) Cross H, 2023 | **Management of Hearing/ Vision Impairment** | Staff working in privately owned homes reported providing hearing support to fewer residents with dementia that they thought would benefit (Mean=49.1) than those working in local authority homes (Mean=57.5), Cross, 2024 | Most managers (>90%) reported that their facilities discussed sensory needs with those affected and their families, Leroi, 2021 | What I've learned from hearing aids is just picked up from other staff members or the nurses. Cross H et al., 2023 | One participant described the sharing of information from ophthalmologists or audiologists to nurses at their facility as "not really communicated very well" [H2N5]; "It can be improved. The reason why because sometimes there's a little bit of a disconnect in the clinic. Because there are still some doctors [...] I don't think they are required to input their data in our electronic system. So they use the handwriting. [...] sometimes it's missed, what's in there or they just write it in but they never tell us that there's another follow-up, unless you really look and read it. So it's still... it's a work in progress." [H2N4], Hobler F et al.,2018. | **Communication** | H1N2: "I don't do hands on with each residents and stuff, so I rely to my staff like the PSW who is there every day taking care of them, doing care with them. So it's just pretty much, I would say, communication has always had to be the key.", Hobler F et al., 2018
**Communication**	Resident's response is unreliable - 2	**Screening for hearing or vision impairments** | Impaired communication can mean not knowing if a client's answer is reliable; many screening tests rely on self-report; In the more advanced stages of dementia, it's very difficult to get a pure-tone audiogram, because pure-tones become meaningless, so you have to use more meaningful stimuli. (participant 2 - Audiologist); not being able to use traditional means of communication or not being able to communicate at all when the neurological condition is too advanced. Wittich W et al.,2018
**Confidence**	Staff lack confidence in recognizing HI - 2<br>Staff lack confidence in recognizing HI/VI with cognitive impairment- 2<br>Staff lack confidence in recognizing VI - 2<br>Not confident in sensory care management - 2<br>Staff confident in incorporating hearing/vision support needs in management plans - 1<br>Confident in supporting people with dementia to use assistive devices - 1<br>Not confident in handling/checking HA - 7<br>Staff lack confidence due to knowledge to manage/handle/check HA - 5<br>Staff confident in providing hearing care - 2<br>Staff confident in providing eye care - 2<br>not confident in handling/checking glasses - 1	**Screening for hearing or vision impairments** | Less than 25% strongly agreed that they were confident in recognising various hearing (17.5%) and vision conditions (8.3%); (Staff) strongly agreed that they were confident in assessing whether a resident has a hearing (24.8%) and vision impairment (14%); strongly agreed that they were confident in assessing hearing (21.5%) and vision impairment (14.5%) in the cognitively impaired., Andrusjak W et al., 2021 | **Use of Device** | (Staff) felt confident that they could incorporate hearing and vision assessments into the care management plans of residents, with managers feeling most confident (86.5% agreed or strongly agreed), followed by nurses/AHPs (75.6%) and care staff (45.6%); The confidence of nonmanager staff in supporting Resident with dementia to use assistive devices (ie, hearing aids, TV amplifiers, glasses, additional lighting) was high, with more than 75% in both groups reporting they agree or strongly agree (managers were not asked this question as this task was considered beyond their role). Leroi I et al., 2021; | a substantial minority (majority of Koreans) reported not knowing how to incorporate hearing/vision needs in management; Korean respondents and a substantial minority of those from other countries reported not being confident in helping residents with use of assistive hearing/vision aids, Dawes P et al, 2021
**Device Comfort**	Aids not tolerated - 2<br>Fitting or usage related issues (causes background noise) - 14<br>Glasses not comfortable - 2	**Use of Device** | "People with a dementia do not tolerate wearing objects that do not fit comfortably/cause irritation".Cross et al. 2023 | discontinued because "spectacles due to discomfort. (Marmamula, Bhoopalan, et al., 2023 | Reason why the patients did not use a hearing aid effectively. Kwak at al. 2022 | 45% responded that the reason individuals did not use their hearing devices was due to poor fit. Leroi et al.,2021 | Pain: experienced discomfort relating to pain. Moroe at al. 2019
**Device management**	Self-capacity to take care of HA - 1<br>Resident's need support to wear/handle/manage HA - 3<br>Residents face challenges in handling/ checking/ maintaining HA - 10<br>challenging to adapt to using communication modalities - 1	**Use of Device** | (Resident not using Hearing aid) "hard to use" (27.60%), "resident forgets to use them" (20.70%). Cross H, 2024 | 20.8% of their residents are able to take care of their own aids, Andrusjak W et al., 2021 | Difficulty with function and maintenance: Five participants (residents) reported difficulty with function and maintenance of their hearing aids.; Hearing Aid Placement: reported difficulty with hearing aid placement; Working the controls: P8 (resident) expressed difficulty regarding the controls on the hearing aid; Cleaning the hearing aids: Participant nine reported his struggle with cleaning the hearing aid, Moroe, 2019; The majority of hearing aid wearers (80%) needed assistance putting in their hearing aids while 91% needed assistance with maintenance tasks, including changing batteries and cleaning. The remainder were carried out either by care home staff, GP practice staff or private practitioners. No details were obtained about the nature of these assessments. White J et al., 2021

Factor	Name of Code - number of times it was coded	Quotes / Verbatim extraction from articles
**Emotional Distress**	Staff are frustrated with hearing care professionals -1<br>Feels frustrated using information and communications technology to communicate -2<br>Residents with HL recognize the frustration experienced by their interlocutors - 1<br>Emotional distress - 1<br>Negative mental emotions of seeking off site hearing care - 1<br>Pessimistic about arranging appointments - 1	**Receiving hearing or vision care** | We'll then ring... the audiology department to explain "the individual that we're dealing with has severe dementia, is there any chance you can come and perform the hearing test here? because if we took them to a hospital, it's a very scary environment and they might not understand what's going on"... We can't really send carers all the time because it then impacts the rest of the residents. (Care Assistant-1, 8 years in profession); It's just that lack of support and feeling alone when having to deal with situations like this. We can put as much stuff into place as we can to make everything easier, but we're not experts in this field... You kind of get to the point where you're like "what is the point?" - (Care Assistant-1, 8 years in profession) - The working relationships with audiology often left LTCH staff frustrated. Cross H, 2023 | **Communication** | the majority of participants expressed frustration in different ways at their attempts at communicating with their family members through ICTs; "I keep saying, 'Say that again, say that again.' So, I'm sure that phoning me is not a joy for him.... My son in England doesn't phone me too often because it's frustrating you know."; "I don't know how to use it. I don't know how to use it and I feel like an idiot. And I wouldn't know to send (sms)". Andrade et al., 2022
**Family Engagement**	Family member reports hearing loss - 1<br>Family member refers - 1<br>Family member does not support hearing services - 1<br>Family member do not consent - 1<br>Family member do not cooperate - 1<br>Family member support HA usage - 3<br>tenacious relatives - 1<br>Family member provide hearing support - 1<br>Family member provide communication technique - 2<br>Family member assist - 2	**Screening for hearing or vision impairments** | The facility managers responded that the reports of family members - most frequently used methods for hearing (screening) tests. They (Facility Managers) conducted their practices based on the reports from family members (30.25%). Kwak C et al., 2022 | **Referring** | They conducted their practices based on the reports from family members (30.25%), White, 2021 | **Receiving hearing or vision care** | We made the decision not to take her to any hearing tests because it would just be pointless [...] She wouldn't cope. She would definitely be stressed... She just wouldn't know what was going on. (FC-2)., Cross H et al., 2024 | non-consenting family members in 49 (16.4%), Mamamula S et al., 2022 | **Use of Device** | I leave notes on the near the HA box. Gradually staff has probably passed it onto new staff to say that her HAs have to come out at night and to open the battery case so that they're disconnected. (Family). Bott A et al., 2022 | Just the loss of the hearing aid, it can create havoc. Especially if you've got very tenacious relatives. (Care Assistant-3, 1.5 years in profession), Cross H et al., 2023 | **Communication** | I leave notes and I tell staff, and if I'm there and they're speaking to her, and I can tell Mum's not picking up the conversation, I just say, "You'll have to speak a bit louder," or "Come closer." (Family). Bott A et al., 2022
**Fellow residents' support**	Fellow residents support use of information and communications technology - 2	**Communication for Hearing Impairment** | "[Fellow resident's name] here is friends with my daughter on Facebook and she shows me the picture." ; "There's a lovely lady here, [fellow resident's name], she lets me email. She has said to me if you have any friends or family that you can't hear over the phone, tell them to email me." , Andrade et al.2022
**Functional device availability**	Aids lost or broken - 1<br>LTC provides information on sensory support available - 1<br>HA lost or broken - 4<br>Alternative communications tools for hearing used- 2<br>Basic assistive/communication devices are available in LTC - 5<br>Lack of assistive devices in resident's room - 1<br>Glasses not available - 1<br>Glasses broken/lost - 1	**Hearing/ Vision Management** | The most common reasons given for Resident with dementia not effectively using hearing or vision devices were "lost or broken."; Most managers informed residents of services and resources available for sensory support (80%). Leroi, 2021 | " did not use them effectively" because "lost or broken"/ "hard to use"/"not effective", Dawes, 2021. | They usually used a (voice amplifier as the test and/) or communication tools with patients. Kwak et al., 2022 | The most commonly used assistive listening devices were loop amplifier (telecoil) systems, which were used in communal areas (29% of homes)., In 47% of participating care homes, these devices(assistive listening devices and other aids to support communication) were not provided within residents' own rooms. White P et al., 2021 | Reason for not using glasses: Broken, Yekta et al., 2019
**Infection control**	Restricted hearing support/management due to COVID - 1<br>Restricted communication facility due to COVID - 2<br>Face mask wear hindered communication - 2	**Receiving hearing or vision care** | Moreover, existing audiology appointments for hearing tests, check-ups and hearing aid maintenance were disrupted due to additional precautions in place for LTCH, Cross H et al., 2024 | **Communication** | Conversations are just so difficult, bordering on impossible now [due to face masks]. (FC-2); The mask thing is just so bad... I try, you know, outside and I say to her, I repeat it. And if she still doesn't get it, I pull the mask down and just say. And she gets it because she can see my lips moving and she can hear better. (FC-6, 71 yrs), Cross H et al. 2024
**Infrastructure**	Lack of screening tools/facilities - 1<br>limitations of portable equipment - 1<br>Lack of visual resources in LTC - 1<br>LTC environment are sensory friendly - 2<br>Lack of dementia friendly service - 1<br>Familiar environment for testing - 3<br>Noisy Environment - 6	**Screening for hearing or vision impairments** | Survey Q: The care home use screening tools to identify hearing/vision loss: 46% of participants stated that they did not use screening tools for hearing assessment; and 43.8% not using vision screening tools. Andrusjak W et al., 2021 | limitations of portable equipment often necessary for these on-location screenings. Wittich W et al., 2018 | 8.7% of LTCFs have a room equipped for eye exams on-site, Keargoat 2014 | Receiving hearing or vision care | complications in arranging transportation for residents with mobility problems, and residents' distress when visiting unfamiliar settings, Cross H et al,2024 | They [audiology department] always want the resident to go to the hospital to have the hearing test. And that's not always possible, especially if you've got someone that has got dementia who doesn't do well with going outside in new environments, a noisy environment. – Manager (11 years in profession); | **Communication** | I think we should always have visual aids. I must admit, finding or trying to . . . Yeah, pretty much finding anything in an age care facility that has actual visual and printed off and labeled, you know is very rare. (Care Staff)., Bott A et al., 2022 | Facilitative communication strategies consisted of environmental cues that helped engage residents who have dementia. Hobler F et al., 2018; | " It's harder if there's people talking and a lot of background noise."; In the survey, 30% [16] thought that having music on at mealtimes (the main time when communication was possible) helped people to relax and 28% [15] disagreed with the statement " There is too much background noise here. " Pryce et al., 2013.
**Knowledge**	Staff lack knowledge in HI - 1<br>Staff acknowledged that residents do not communicate if hearing loss is present - 2<br>Hearing loss is not recognized as a health issue - 1<br>Staff lack knowledge or ability to identify SI - 4<br>Job title "type of professional role in LTC"-1	**Screening for hearing or vision impairments** | Most participants reported lacking knowledge of hearing loss, Cross H at al., 2023 | Some participants thought a combined vision and hearing loss involved no hearing or vision at all. (lack of understanding), Wittort, 2023 | Among front-line nurses and care workers (groups 2 and 3), approximately 46% of the 2 groups combined reported insufficient training to administer or interpret such tests (disagree or strongly disagree), Leroi, 2021 | H2N4: "...from my experience I've never conducted an official tool. Like it's just basing on your nursing assessment, let's say, the changes in behaviour, changes in the routine, but we don't have the tool, let's say, for the pain scale. We have a tool for skin assessment; we have a tool to use, but we don't have for the hearing and for the visual."; Hobler, 2018. | They identified hearing loss as the primary basis of communication difficulties when the residents did not respond to them. Slaughter S at al., 2014 | What is

Factor	Name of Code - number of times it was coded	Quotes / Verbatim extraction from articles
	Lack of awareness of dual sensory impairment - 4<br>Staff manage/handle/check HA - 9<br>Staff lack knowledge to manage/handle/check HA - 10<br>Staff have knowledge about HA - 2<br>Family member unsure of alternative methods of management - 2<br>staff to understand the implications of adjusting to amplified sound-1<br>Learned by experience - 1	striking is the recognition that a hearing loss presents a shared communication problem rather than being regarded as an individualized health issue, Pryce H., at al, 2013 | **Use of Device** | Participants (Family members) were unsure of the best approach to support their relatives' hearing due to residents' difficulties with traditional hearing aids; Most were unaware of alternative methods. Cross H et al., 2024 | Participants perceived they did not have sufficient skills or knowledge to adequately support individuals with sensory impairment, especially those with combined vision and hearing loss: "I don't really know what I'd do. (Personal care assistant, aged 20 years); I'd probably just talk to the OT [occupational therapist]....Um, I'd probably kind of hand ball that to her. (Facility manager, aged 53 years)", Wittorff, 2023 | The job title was also a significant predictor of behaviour (Registered nurses reported providing hearing support to more resident than carer), Cross H et al., 2024 | Participants perceived they did not have sufficient skills or knowledge to adequately support individuals with sensory impairment, especially those with combined vision and hearing loss: "I don't really know what I'd do. (Personal care assistant, aged 20 years); I'd probably just talk to the OT [occupational therapist]....Um, I'd probably kind of hand ball that to her. (Facility manager, aged 53 years)", Wittorff M at al., 2023 | Most participants reported lacking knowledge on how best to manage residents' hearing difficulties, placing emphasis on their variable knowledge of hearing aids. Cross H et al., 2023 | But how we are managing it here, is that we keep them [HA] in their case and kept in the medication trolley. That's what I've implemented here, similar to what I implemented at the other place, because it's very costly. So, I make sure that the RN puts the hearing aids on in the morning, because it's on the medication trolley. The evening shift RN, before going out . . . During their 8:00 medication rounds at night, they take the hearing aid off and put it back in the trolley. (Care Staff), Bott A, 2022 | lack of education in checking and/or cleaning hearing aids (17.22%), in turning hearing aids on/off (10.60%), in checking to see whether hearing aids were working or not (4.42%), in changing the batteries of a hearing aid (1.32%), Kwak C et al, 2022 | Twenty-nine percent believed they had adequate knowledge about the residents' hearing aids; 35% of staff were unable to provide the necessary assistance; 64% of the informants regularly checked the battery's + side before inserting it into the hearing aid; only 29% were familiar with the approximate lifetime of a hearing aid battery. The informants considered themselves able to provide hearing aid assistance to varying degrees 35% were unable to provide the necessary assistance., Twenty-nine percent believed they had adequate knowledge about the residents' hearing aids, Solheim J et al., 2016 | there is a need for staff to understand the implications of adjusting to amplified sound. Pryce H et al., 2013.
**Logistics**	Long wait times - 1<br>Multiple visits required - 1	**Receiving hearing or vision care** |When they [resident] come here, we straight away call the GP to refer the audiologist and sometimes it's quick and sometimes it takes time. Months even. Not weeks... (Senior Carer-1, 17 years in profession), Cross H, 2023 | A referral would involve multiple visits for the resident and carer to the GP and audiology department., Pryce, 2013
**Meeting Expectations**	HA did not meet the expectations - 1<br>Resident's satisfied with HA - 1<br>Hearing care professionals did not meet expectations while dispensing HA - 2<br>Feels satisfied with use of information and communications technology - 1	**Use of Device** | the majority of the participants felt that their expectations of hearing aids were not met by audiologists; Moroe et al. 2019 | Almost one-third were of the impression that residents were satisfied with their hearing aids. Solheim et al.2016 | **Communication** | Only one participant expressed a sense of satisfaction with successful communication, notwithstanding the communication difficulties imposed by her being deaf, when she managed just to hear her son's voice (with use of information and communications technology); Andrade et al. 2022; P8 reported "I have a friend and he's got a tiny little thing that fits right in the ear and that is what I've been trying to get and the agent for South Africa has a firm in Edenvale, I can give you the name. It's a tiny little thing, doesn't fit over the ear, it goes right inside the ear. I didn't know there were different types to choose from. I was not told about the different options". P3 stated "Well, they didn't even give us an option, it was 'this is the one we have' and that was it."., Moroe et al., 2019
**Mobility**	Independent Mobility - 1<br>Bedridden - 2<br>Assisted mobility -1	**Receipt of Vision Care** | Idependent mobility - the uptake of referral services was highest (59.1%) among those who were independently mobile, compared with those who needed assistance or were bedridden (p<0.01). The uptake of services was higher in subjects who were independently mobile (OR 5.74; 95% CI 3.13 to 10.51); The uptake of services was higher in subjects who were independently mobile (OR 5.74; 95% CI 3.13 to 10.51) or those mobile with assistance (OR 3.65; 95% CI 1.96 to 6.80) compared with those who were immobile/bedridden; (some of the major reason for not availing the referral services(Residents not attended N = 356) health issues in 100 (33.4%) participants, Marmamula, Kumbham, et al. (2023)
**Onsite service**	Lack of onsite or nearby Hearing service - 6<br>Hearing/Eye care professionals deliver onsite hearing/vision exam - 4<br>LTC provide onsite testing - 2<br>Lack of onsite or nearby vision service - 1<br>Availability of onsite eye examination room/facilities - 1	**Receiving hearing or vision care** | It always seems to be quite a fight to get them to do a home visit instead of them [resident] going to the hospital. I don't think they understand the logistics of trying to get a resident to the hospital. Cross H et al, 2023 | Audiology appointments took place in the community (48.7%), Cross H , 2024 | Regular contacts to hearing aid acousticians (44.3%) and optometrists (32.6%) were less common., Schroder A,2020 | The majority of managers reported that their facility did not provide in-house hearing testing (78%) and vision testing (59%), Leroi, 2021 | In 75% of cases, a resident requiring a hearing assessment would have to travel to another location for assessment., White, 2021 | Aged care staff : "We're very fortunate here [...] that everything is just in the facility. So, they don't have to go out and families don't really feel like 'Oh, my gosh, I have to be there for this assessment' [...], then they're more inclined to say okay, go ahead, do the assessment, whatever you want.", Hobler, 2018. | 8.7% of LTCFs have a room equipped for eye exams on-site; 31.8% have an optometrist, 12.4% an ophthalmologist, and 26.9% an optician, delivering services on-site; some LTCFs have access to an optometrist (54.3%) or optician (41.9%) on-site on a regular basis; Keargoat, 2014
**Onus of responsibility**	Staff/family member feel responsible for fixing an appointment with hearing care professionals - 2<br>Family member consider staff responsible - 2<br>Feeling Responsible for the Provision of Hearing Support - 7<br>lack of Personal Accountability for Hearing care - 2<br>Staff felt responsible to facilitate communication - 2	**Screening for hearing or vision impairments** | I (Facility manager) feel a bit responsible for putting a bit of pressure on audiologists, saying "Hiya. I really need you to come and see this lady that's delusional, she's hallucinating, she's going through all this stuff" so I feel that that's me. (Manager, 11 years in profession), Cross, 2023 |I've (Family member) always taken over really. So, you know, I've gone in and even though I work full-time, before COVID... it was me that took her [to audiology appointments]. If I was really busy, then they [staff] would do it. But you know, I wanted to take her, in a way. (Family Carer-2), Cross, 2024 | **Hearing/ Vision Management** | Only 15.3% of participants saw themselves as responsible for supporting residents' hearing, with care staff (35.2%) and nurses (35.2%) considered equally responsible., Cross H et al., 2024. | It's part of residents' care, isn't it? And if you're not doing it, we're falling short, aren't we? So yes, it is a nurse's responsibility. (Nurse-1, 23 years in profession); I think staff need to take more of an onus on the responsibility for the hearing aids and whose job role it is, rather than just letting the resident try and find their own hearing aids. (Therapy Assistant, 1.5 years in profession) Cross H et al., 2023 |**Communication**| They also considered themselves responsible for social contact between residents " You ' ve got to be vigilant really. " (to avoid communication breakdown). Pryce C et al., 2013.

Factor	Name of Code - number of times it was coded	Quotes / Verbatim extraction from articles
**Perception of values**	Perceived need for screening -2<br>Perceived benefits of screening - 1<br>Sensory screening is acceptable for people with dementia - 2<br>Staff perceived impact of HL & VI on Quality of Life - 3<br>People with dementia would benefit from Sensory support - 2<br>Perceived benefits of HA usage - 6<br>Perceived importance of HA usage - 4<br>Resident feel HA is not beneficial - 6<br>Perceived negative consequences of not receiving hearing support - 2<br>HA alone not sufficient -1<br>Staff perceive HA as not beneficial - 1<br>Glasses do not improve vision - 1<br>Glasses were not useful - 2<br>Vision aids not effective - 2<br>lack of felt need - 1<br>Perception that residents could not cooperate - 1<br>Perception that the eye conditions could not be treated - 1<br>apprehension about traditional hearing aids for people with dementia - 3<br>HA not appropriate for people with dementia - 2<br>staff value effective communication - 1<br>Perceived benefits of communication strategies - 2<br>Perceived importance of communication by Staff - 1<br>HA not helpful in communication - 2	**Screening for hearing or vision impairments** | Most respondents in all 3 groups (agreed or strongly agreed, >68%) felt that brief hearing and vision screens would be acceptable to residents with dementia, Leroi, 2021 | "most respondents agreed hearing/vision screening would be acceptable to residents", Dawes, 2021 | H2N8: "You know, as I said it's just the key element is timing, really; [...] and making sure things are done in a certain amount of time in between so you catch, so things don't deteriorate quickly on you.", Hobler, 2018 | **Receiving hearing or vision care** | I think there'd be more choice... we would help the residents to feel heard, which for me, that's just crucial... I very often see that people don't get relief until they get heard. Whether that's their emotions, their thoughts, their feelings... (Mental Health Nurse-1, 26 years in profession) Cross et al., 2023 | ; | "lack of felt need" reported by 136 (45.4%)- Lack of felt need' was more among those (residents) referred for surgery compared with non-surgical referrals (60.6% vs 41.2%; p=0.01), Marmamula, Kumbham, et al. (2023)| **Use of Device** | But until it's [hearing] taken away from you, you don't realise how much it has an impact on everything that you do... The joy of listening to music, people really take it for granted, but if you can't listen to music, then the emotion has gone. (Manager, 11 years in profession), | With hearing aids, like they can die, they can get lost, they're not that reliable, whereas communication cards are quite... they're just easy and they're quite accessible. (Therapy Assistant, 1.5 years in profession). Just for residents wearing them [hearing aids], for some of them it possibly is just the stress of having them put on, if they don't like to be touched, can be more of a hindrance than actually the benefit of actually being able to hear better. (Nurse – 1, 23 years in profession). Cross H et al., 2023 | 8 (16.7%) reported that they no longer found their spectacles to be useful.. (Marmamula, Bhoopalan, et al., 2023| "It's more clear without the hearing aid over the phone." (resident) ; "I take it out. I can't use it on the telephone, definitely not" (resident), Andrade et al., 2022 | nearly all managers agreed or strongly agreed that the same proportion felt that improving hearing and/or vision functioning would benefit resident with dementia cognitive and functional ability., Leroi, 2021 | Of 55 participants who had prescription glasses, 28 did not wear them regularly mostly because wearing them did not improve their vision (50%). Yekta et al., 2019 | The most oft-cited concern of nurses in relation to residents in their care who had vision or hearing loss, as well as cognitive impairment, was the impact of these challenges on their quality of life. Hobler F et al., 2018 | " There's a few people that are really hard of hearing although they do have their hearing aids in. " (Mary - staff). Pryce H et al., 2013 | **Communication** | "sometimes, even with hearing aids, it is difficult to communicate.". Cross H et al., 2024 | effective use of communication strategies improved residents' mood, energy levels and engagement in conversations. Bott A et al., 2022 | Staff valued communication and considered communicating with residents to be an important part of their work. Pryce H et al., 2013
**Prioritization**	Staff do not prioritize sensory screening - 1<br>Hearing care is overlooked - 1<br>Staff prioritized hearing care - 1<br>Hearing loss had low priority due to systemic ailments - 2	**Screening for hearing or vision impairments** | Participants speculated that this absence may, in part, be due to policies or regulations that do not include or promote sensory screening, lack of education and training on how to screen or not prioritising hearing and vision screening given the multiple demands on their time, Wittich W et al., 2018 | **Hearing/ Vision Management** | "She complains about things, and then she does say her ears are sore sometimes, or she can't hear, then she goes onto something else... it's difficult. (FC-5, 61 yrs); ...Having her ears syringed is a low priority thing. (FC-3, 53 yrs)"; Food, yes, drink, stopping people from falling. You know, there's all these things aren't there? And so, you know, if you can get by...by shouting and using a pen and paper, then... it [hearing support] will go down the list. (FC-1, 58 yrs). Cross H et al., 2024. |When asked whether hearing loss was a high priority compared to other care needs, 68.4% responded >7 on a 0–10 scale. Cross H et al., 2023/24 | (Staff) reported hearing support to be "everybody's job", this was not always productive, as hearing can be easily overlooked. Cross H et al., 2023
**Protocols**	Recorded/ Referred in Resident's report - 2<br>Hearing NOT tested on admission - 1<br>Hearing tested on admission - 1<br>Eye health is documented at entry level - 1<br>Lack of entry-level eye screening - 1<br>Sensory screening process is not systematic - 1<br>SI is documented - 1<br>Systematic Sensory Screening - 3<br>LTC provides information on sensory services - 1<br>Referred to professionals if concerns arise during screening - 1<br>LTC provide access to hearing care professionals - 1<br>LTC refer to audiologist - 1<br>LTC refer to ear hospitals - 1<br>Resident's are assessed by Health practitioner - 1<br>Resident's are assessed by audiologist - 1<br>Resident's are assessed by care staff - 1<br>LTC Coordinate appointments with Eye Care Professional - 1<br>Referred to GP - 1<br>LTC managers provided referral pathway - 1	**Screening for hearing or vision impairments** | The facility managers responded that the reports of patients - frequently used methods for hearing (screening) tests., Kwak C et al.,2022 | When they [resident] come here, we straight away call the GP to refer the audiologist and sometimes it's quick and sometimes it takes time. The majority (86%) of care homes reported that, should an existing resident experience hearing difficulties, their GP would be contacted. In 9% of care homes, the resident would be referred directly to their local NHS Audiology service; While 88% of respondents reported that their care home did not routinely assess residents' hearing on entry to the home. White J et al., 2021 | Each facility had a designate member of nursing staff who was responsible for screening residents upon admission, at regular intervals (quarterly and annually), and tracking when residents were due to be screened again.., Hobler F et al., 2018 | The alternatives (for hearing screening) included referral to hospital, Kwak C et al., 2022 | 72.2% do not offer any formal oculovisual screening. Keargoat H et al., 2014 | **Hearing/ Vision Referral** | Most managers informed residents of services and resources available for sensory support (80%)., Leroi I et al., 2021 | The majority (86%) of care homes reported that, should an existing resident experience hearing difficulties, their GP would be contacted. White J et al., 2021 | **Hearing/ Vision Management** | A champion in the care home that they trained up... and everybody in the care home knew this carer or this nurse is the person that knows about hearing aids, and any questions that they've got they can refer to them. (Nurse-1, 23 years in profession). Cross H et al., 2023 | Only 30.9% had a specifically designated staff member responsible for hearing support in their place of work. Cross H et al., 2023 | Finally, more than 40% of front-line staff (nurses, AHPs, and care staff) stated that they did not carry out regular checks or testing of hearing and vision aids. Leroi et al., 2021;

Factor	Name of Code - number of times it was coded	Quotes / Verbatim extraction from articles
	Designated staff for hearing support - 1<br>Staff do not regularly check HA/ VA - 2<br>Lack of designated staff for hearing/ vision care in LTC - 4<br>Sensory care plan documented - 1	
**Staff Engagement**	lack of staff involvement - 1	**Screening for hearing or vision impairments** | Lack of staff involvement in the screening of persons for hearing and vision loss, Wittich, 2018
**Time commitment**	Staff have time constraints in accompanying residents for off-site test - 2<br>Staff accompanying residents leaves other residents wither fewer carers - 1<br>Lack of time to attend scheduled training - 1	**Receiving hearing or vision care** | Transportation for residents with a staff member was difficult, as accompanying a resident to an appointment means being away from the LTCH for several hours, potentially leaving other residents with fewer caregivers., Cross H, 2023 | This involve arranging [....] and considerable time for the staff., Pryce 2013 | Use of Device | I couldn't make it (hearing mob - training in aged care) cause I was on the floor [providing direct care], Wittorff, 2023
**Training**	Family member wants more information on hearing support - 2<br>Limited Training Opportunities - 9<br>Staff need more clinical guidelines for management - 1<br>Staff want more information/training about HA - 5<br>Staff want to know more screening techniques-2<br>Staff are given training on HA - 1<br>Staff need more information/training about HI - 2<br>LTC provide staff training for hearing management - 1<br>Staff need more information/training about VI - 1<br>LTC provide staff training for vision Management - 2<br>LTC provide communication training - 1<br>Staff to be trained to use communication strategies - 4<br>Lack of formal communication training - 1<br>Residents were not given information on HA usage -1	**Screening for hearing or vision impairments** | Of the group 1 professionals (managers), a quarter to a half disagreed or strongly disagreed that they had sufficient training for using such vision and hearing screening tools, respectively., Leroi I et al., 2021 | The knowledge and skill development required to carry out sensory screening was not reported to be currently offered or instructed through educational provision at either facility. Throughout the interviews, nurses expressed their desire to learn more about tools and strategies that can be used to better screen residents in their care. Hobler F et al, 2018 | **Hearing/ Vision Management** | About 63.2% wanted to know more about how they can support their relatives' hearing. Although all wanted to know more about how best to support their relatives' hearing, they were unsure of where they could access information specifically for supporting hearing loss in people with dementia. Cross H et al., 2024 | Every participant revealed a lack of training on hearing loss and hearing care/support within their workplace. What I've learned from hearing aids is just picked up from other staff members or the nurses. We don't really have any kind of formal training, or anything that I can think of where I could refer to... like any company policy kind of thing to say this is what we do with hearing loss. (Therapy Assistant, 1.5 years in profession), Cross H et al., 2023 | Six participants indicated that they were not provided with adequate information on how to care and operate their hearing aids. Moroe et al.2019 | Most (75%–98%) staff reported not having training and support to use sensory equipment., Dawes P et al., 2021 | The most common reason for the nonmanager respondents' lack of confidence in providing sensory care was lack of training on the use and maintenance of the hearing and vision devices; Front-line staff - the majority reported that they did not have the training necessary to do this (check and test hearing aids) (>70%). Most respondents in all 3 groups the majority felt that clinical guidelines for hearing and vision care for this resident group would benefit their ability to deliver good sensory care (agreed or strongly agreed, 60%, 79%, and 76% in groups 1, 2, and 3 respectively) Leroi, 2021; | H2N2: "It can be improved, like, staff education ... should be like tell staff how to, like, take care of the hearing aid better like handling wise, sometimes you have to teach them. [...] some of them they bring new hearing aids from where we don't know it's - it's high tech. So - but they always have better education is always good; like the staff needs to be just you know, reminded." Hobler F et al., 2018 | Despite their considerable practical experience, nearly half of respondents reported little formal training: 48% [26] of survey respondents reporting that they had not been offered any training in hearing loss. Pryce H et al.,,2013 | **Communication** | These strategies are learned by experience in communicating with the residents rather than through any formal training. Pryce C et al., 2013
**Transportation**	Transportation arrangements - 2<br>LTC offer transport - 1	**Receiving hearing or vision care** | I (Family member) think it's more the difficulty in arranging her to be referred and then getting an appointment [...] there's no way she'd get into my car now [...] (FC-6, 71 yrs), Cross H, 2024. | 93.8% of long-term care facilities offer transportation., Kergoat, 2014
**Value of care plan**	Perceived importance of sensory care plan - 2	**Hearing/ Vision Management** | quality of the care plan is essential in knowing what strategies to use when supporting them., Wittorff M at al., 2023 | Attitude - most respondents agreed "would find clinical guidelines for assessment and management of hearing/ vision useful", Dawes P at al., 2021
**Willingness**	Willingness to use HA - 1<br>Resident don't prefer HA - 6<br>Willingness to use glasses - 1<br>Residents exhibit deliberate behavior - 1	**Use of Device** | We had a year of her [resident] not wearing the hearing aids, because she didn't have a problem, so she says, so she would hide them. (FC-4, 46 yrs). Cross et al H., 2024 | residents are willing to use their hearing aids (25.8%). Andrusjak W et al., 2021
**Workforce stability**	knowing/time spent with resident - 2	**Screening for hearing or vision impairments** | After caring for residents over an extended period of time, one healthcare aide stated that she could differentiate between the impact of hearing loss and dementia on communication, Slaughter, 2014

HA: Hearing aid; HI: Hearing impairment; VI: Vision impairment; VA: Visual aids (glasses); GP: General Physician

Appendix 6: Inter-Relationship Diagrams (IRD) for each behaviour

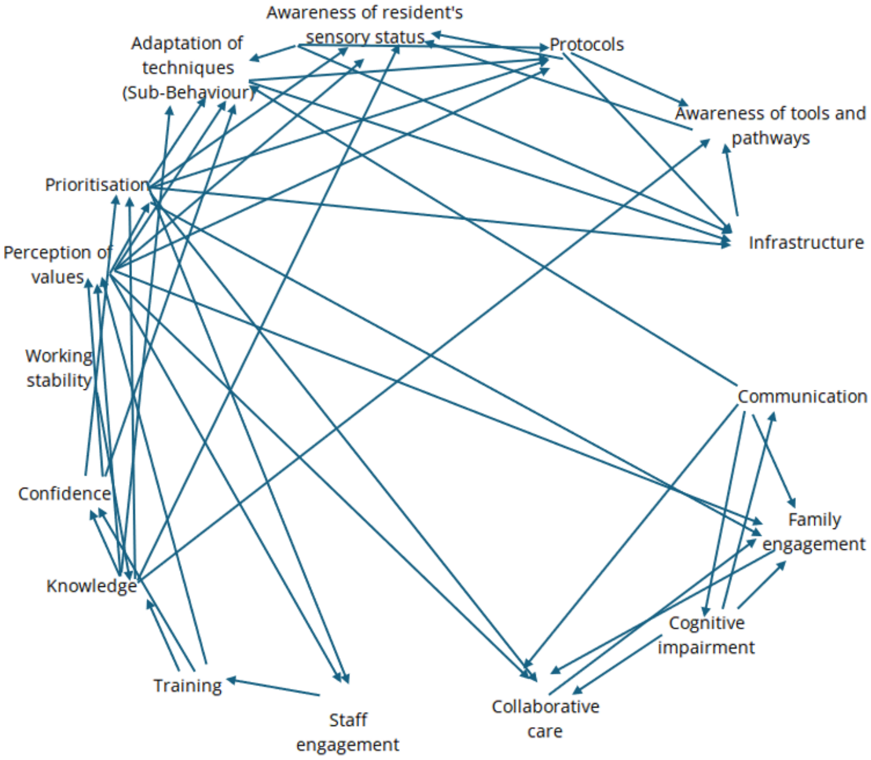

IRD for Screening for hearing or vision impairment

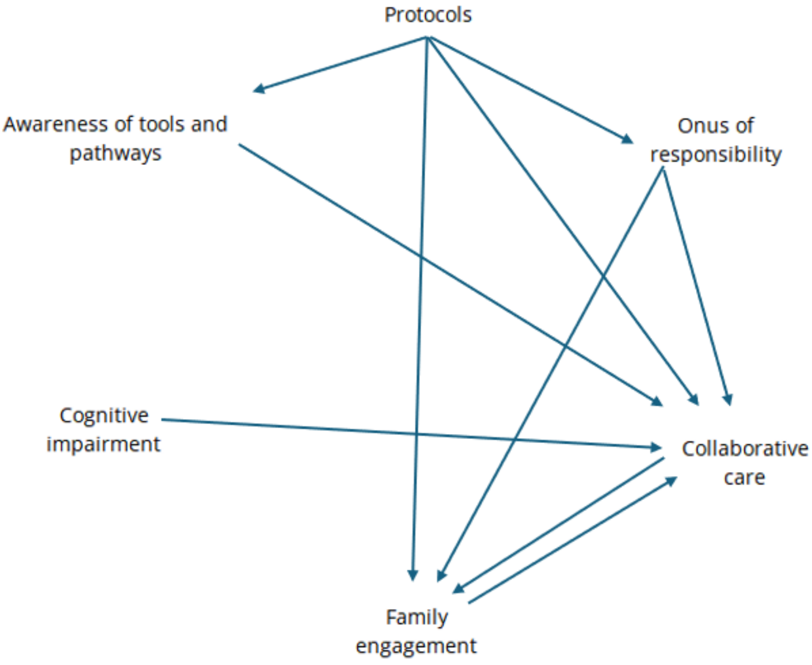

IRD for Referring residents to hearing or vision services

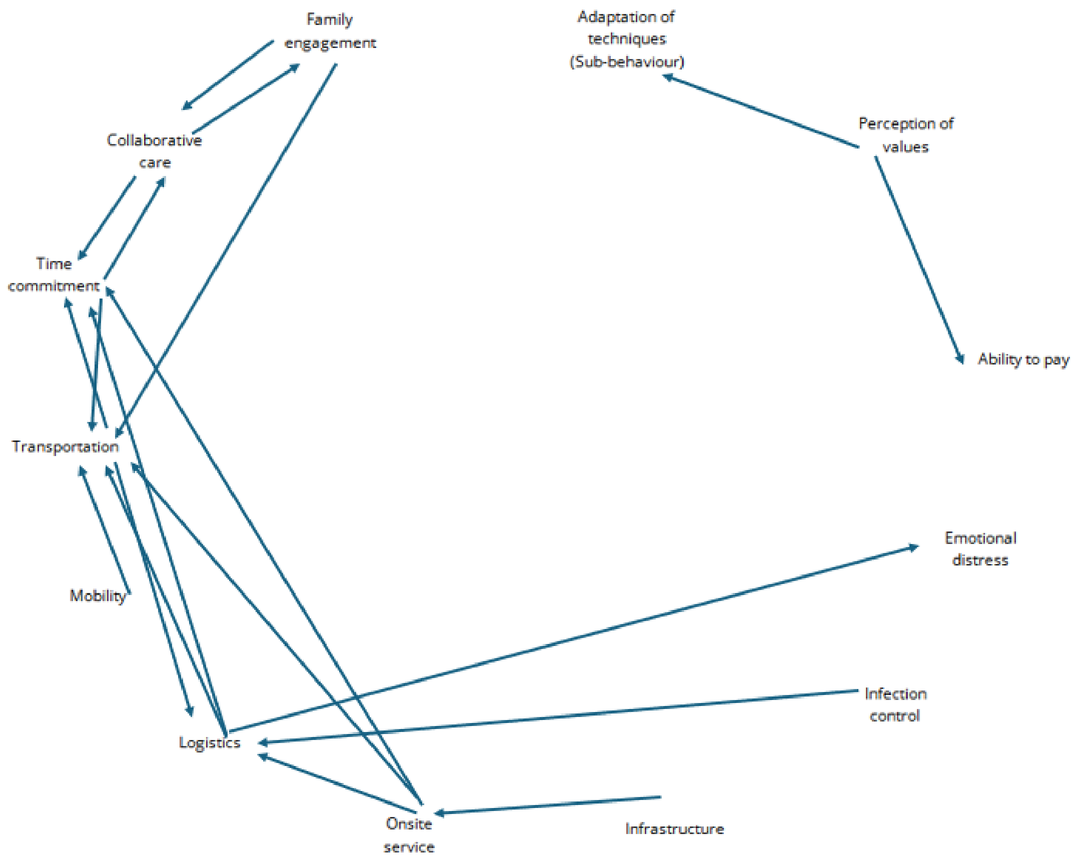

IRD for Receiving hearing or vision care

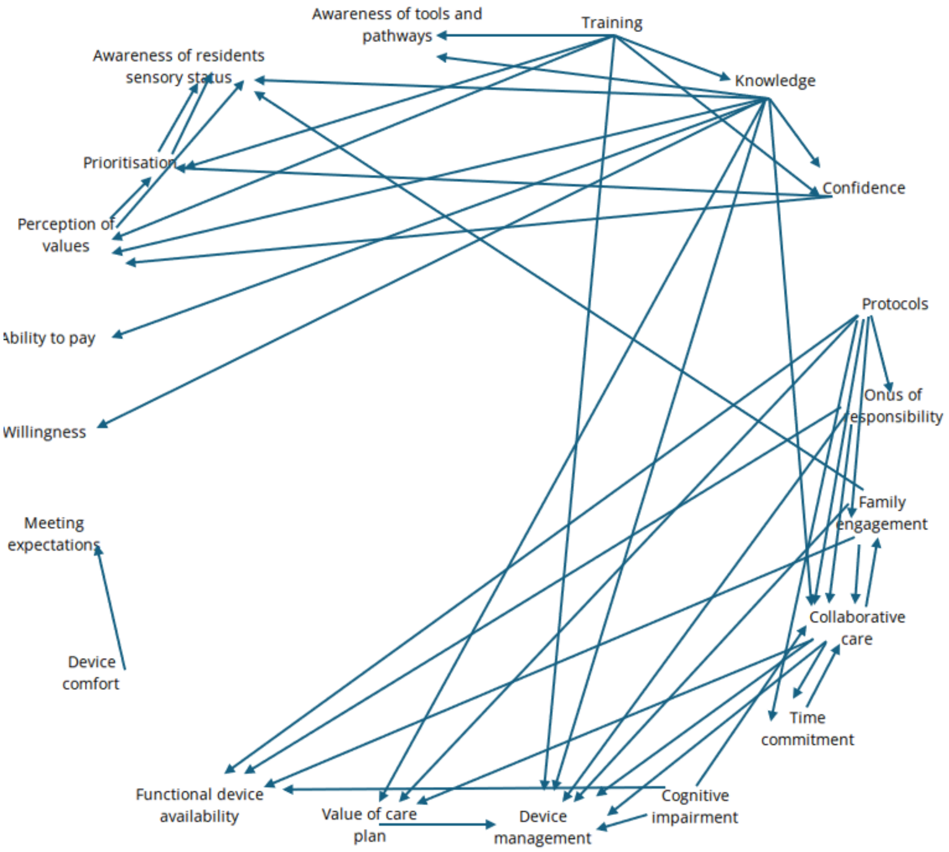

IRD for Use of Device

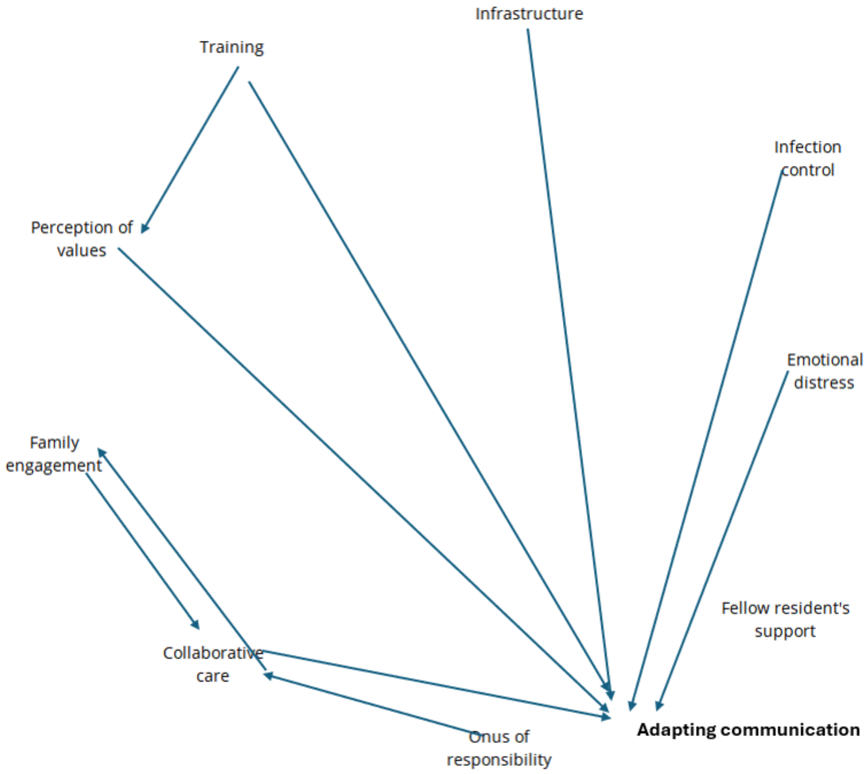

IRD for adapting communication
